# Transcriptome analysis of the effect of *Vibrio alginolyticus* infection on the innate immunity-related complement pathway in *Epinephelus coioides*

**DOI:** 10.1186/1471-2164-15-1102

**Published:** 2014-12-13

**Authors:** Yi-Da Wang, Shin-Jie Huang, Hong-Nong Chou, Wen-Liang Liao, Hong-Yi Gong, Jyh-Yih Chen

**Affiliations:** Institute of Fisheries Science, National Taiwan University, 1 Roosevelt Road, Sec. 4, Taipei, 106 Taiwan; Marine Research Station, Institute of Cellular and Organismic Biology, Academia Sinica, 23-10 Dahuen Rd., Jiaushi, Ilan, 262 Taiwan; Institute of Cellular and Organismic Biology, Academia Sinica, 128 Academia Road, Section 2, Nankang, Taipei, 115 Taiwan; Department of Aquaculture, National Taiwan Ocean University, 2 Beining Road, Jhongjheng District, Keelung City, 202 Taiwan

## Abstract

**Background:**

Orange-spotted grouper (*Epinephelus coioides*) with protogynous hermaphroditic features are one of the most economically important aquaculture species in Taiwan. However, larvae stage grouper are susceptible to infection by the bacterial pathogen *Vibrio alginolyticus*. To better understand the molecular mechanisms of the immune response to *V. alginolyticus* in *Epinephelus coioides* larvae, we used high-throughput deep sequencing technology to study the effect of infection on gene expression.

**Results:**

A total of 114,851,002 reads were assembled, consisting of 9,687,355,560 nucleotides; these were further assembled into 209,082 contigs with a mean length of 372 bp. Gene ontology (GO) analysis of the transcriptome revealed 12 cellular component subcategories, 16 molecular function subcategories, and 42 biological process subcategories (P value <0.05). A total of 32664 *Epinephelus coioides* genes were mapped to the Kyoto Encyclopedia of Genes and Genomes (KEGG); 1504 differentially expressed genes (DEGs) were subsequently identified, in 12 categories (P value <0.05). *Vibrio* infection affected the expression of genes involved in complementation, coagulation cascades, pathogen (*Staphylococcus aureus*) infection, phagosome activity, antigen processing, and the antigen presentation pathway.

**Conclusion:**

We conclude that the complement pathway of innate immunity and the hepicidin antimicrobial peptide may play important roles in the defense of *Epinephelus coioides* larvae against *V. alginolyticus*, and the immune response may activate at 4 h after bacterial infection. These results implicate the complement pathway signal pathway in immunity during *V. alginolyticus* infection at early developmental stages, enhancing our understanding of the mechanisms underlying the immune response to *Vibrio* infection in *Epinephelus coioides*.

**Electronic supplementary material:**

The online version of this article (doi:10.1186/1471-2164-15-1102) contains supplementary material, which is available to authorized users.

## Background

Groupers are an economically important aquaculture species in Southeast Asian countries, with a high market demand in several locales, including Hong Kong, Taiwan, China, Mexico, Japan, and the USA. However, intensive culture of grouper can result in outbreaks of infectious disease, caused by viral pathogens, such as nodaviruses and iridoviruses, or bacterial pathogens, such as *Vibrio carchariae* and *V. alginolyticus*[1]. Grouper zygotes develop by 24 h post-fertilization, and their yolk sacs disappear by 72 h. By 10 days, grouper larvae begin to expand their dorsal and ventral fin rays to form an inverted triangle. At this stage, grouper larvae are easily infected by pathogens. After one month, the long fin reaches its full size, and body shape begins to resemble that of the mature grouper [2]. The orange-spotted grouper (*Epinephelus coioides*) possesses protogynous hermaphroditic features, and is easier to culture than certain other grouper species, such as *Epinephelus lanceolatus*. Furthermore, *Epinephelus coioides* is an excellent source of nutrients; consequently, this species is widely cultured in Taiwan. An earlier study reported that expression of C3 mRNA in *Epinephelus coioides* is influenced by pH and temperature stress, and may play an important role in antioxidation mechanisms [3].

*Vibrio alginolyticus* is a Gram-negative bacterial species with a straight rod shape, and is positive for oxidase and catalase. This bacterial species can cause gastroenteritis, with swelling of the intestine. Furthermore, it is a potential pathogen of marine fish and shrimp [4]. *V. alginolyticus* strain S3y has been isolated from grouper (*Epinephelus malabaricus*) larvae with vibriosis in Taiwan; this strain is a pathogen of particular concern, as it causes enormous economic losses in the aquaculture industry [5]. Next-generation high-throughput DNA sequencing techniques, including that provided by the Illumina Genome Analyzer, provide high speed and throughput (gigabase level). Such techniques can be used to identify and quantify rare transcripts without prior knowledge of gene sequence, and provide information regarding alternative splicing and sequence variation in identified genes; as a result, high-throughput sequencing is more effective at detecting genes than microarrays [6, 7].

In this study, we used high throughput sequencing to identify differentially expressed genes (DEGs) between normal grouper larvae and larvae with vibriosis. The DEGs were classified based on their Gene Ontology (GO) categories and the Kyoto Encyclopedia of Genes and Genomes (KEGG). Furthermore, comparative RT-PCR was used to confirm the observed effects on genes involved in likely pathways affected by infection, to elucidate the molecular mechanisms underlying vibriosis in grouper larvae.

## Results

### De novo sequencing and read assembly of the *Epinephelus coioides*transcriptome following *V. alginolyticus*infection

**Figure 1:**
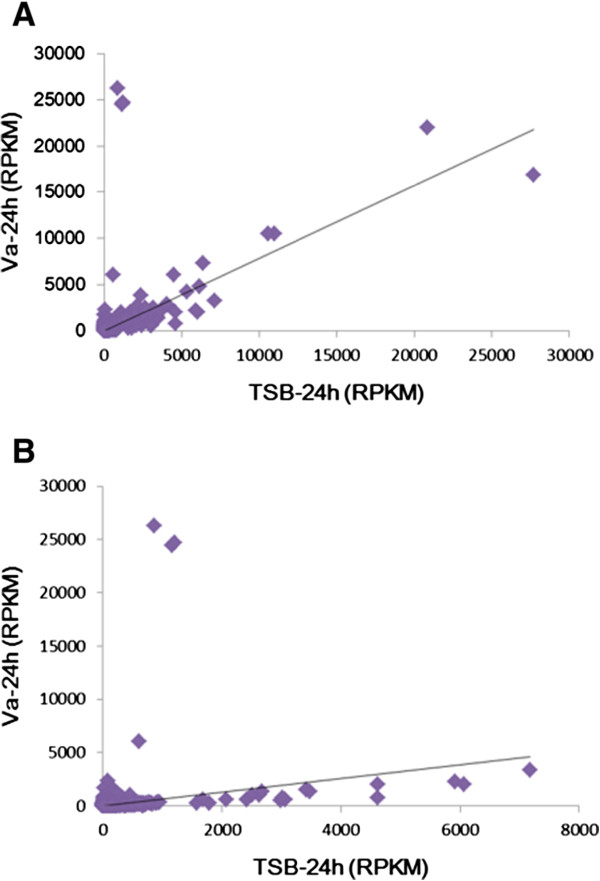
**Comparison of unigene expression as Reads Per kb per Million (RPKM).** Expression level was determined using the RPKM method, thereby eliminating the influences of gene length and sequencing discrepancies on the calculation of gene expression. ***A***, RPKM with 102,857 unigenes, ***B***, RPKM after selection based on FDR ≤ 0.001 AND |log_2_Ratio| ≥ 1with 3,977 unigenes. Va-24 hr: RPKM at 24 hours after infection with *V. alginolyticus*, TSB (tryptic soy broth)-24 hr: RPKM at 24 hours after injection with TSB.

The assembled transcriptome consisted of a total of 114,851,002 reads of 9,687,355,560 nucleotides; these were further assembled into 209,082 contigs with a mean length of 372 bp. The total contig length (nt) was 77,845,532, and 116,678 unigenes were identified, with an average length of 685 bp; the total unigene length (nt) was 79,966,605. Of these unigenes, 69334 unigenes were present in all databases (Table 1). We identified 3977 DEGs (FDR ≤ 0.001 and |log_2_Ratio| ≥ 1) between the control and *Vibrio* challenge group. These include 1104 up-regulated unigenes, and 2873 down-regulated unigenes (>2 fold-change in value). A linear ratio was observed between the RPKM of the *V. alginolyticus*-infected group and the TSB (tryptic soy broth)-injected group after 24 h (10,2857 unigenes) (Figure 1A). After selection of genes with FDR ≤ 0.001 and |log_2_Ratio| ≥ 1 (3,977 unigenes), we observed that gene expression was considerably higher in the TSB group than in the *V. alginolyticus* group (Figure 1B).

**Table 1:** **Summary of** ***Epinephelus coioides*** **larvae transcriptome assembly**

Transcriptome sequences	
Total Reads	114,851,002
Total Nucleotides (nt)	9,687,355,560
Total Contig Number	209,082
Mean Length of Contig (bp)	372
Total Contig Length (nt)	77,845,532
Total Unigene Number	116,678
Mean Length of Unigene (bp)	685
Total Length of all Unigene (nt)	79,966,605
Genes in NR database	53518
Genes in NT database	64066
Genes in SwissProt database	46315
Genes in KEGG database	37075
Genes in COG database	14422
Genes in GO database	18252
Genes in all databases	69334

### Identification of differentially expressed genes (DEGs) via GO and KEGG analysis

Gene ontology (GO) analysis of the 3,977 unigenes was performed using open source clustering software for Annotation [8], and cluster analysis was performed using cluster software and Java treeview software. GO analysis of the transcriptome revealed 12 cellular component subcategories, 16 molecular function subcategories, and 42 biological process subcategories (P value <0.05) (Additional file 1: Table S2). The largest subcategory in the molecular function group was ‘hydrolase activity’, which was represented by 24.9% of the clustered genes. In the cellular component and biological process categories, ‘cytoskeleton’ and ‘small molecule metabolic process’ were the most abundant GO terms, making up 15.3% and 19.5% of each cluster, respectively (Table 2).

**Table 2:** **Gene ontology analysis of** ***Epinephelus coioides*** **larvae**

**Cellular component**			
Gene Ontology term	Cluster frequency	Genome frequency of use	Corrected P-value
Myosin complex	45 out of 1129 genes, 4.0%	162 out of 28794 genes, 0.6%	1.46E-23
Actin cytoskeleton	79 out of 1129 genes, 7.0%	656 out of 28794 genes, 2.3%	1.62E-16
Contractile fiber	59 out of 1129 genes, 5.2%	501 out of 28794 genes, 1.7%	1.28E-11
Extracellular region	106 out of 1129 genes, 9.4%	1483 out of 28794 genes, 5.2%	3.55E-07
Myofibril	43 out of 1129 genes, 3.8%	433 out of 28794 genes, 1.5%	6.02E-06
Sarcomere	36 out of 1129 genes, 3.2%	327 out of 28794 genes, 1.1%	6.05E-06
Contractile fiber part	36 out of 1129 genes, 3.2%	332 out of 28794 genes, 1.2%	8.94E-06
Cytoskeleton	173 out of 1129 genes, 15.3%	3012 out of 28794 genes, 10.5%	3.39E-05
Extracellular region part	92 out of 1129 genes, 8.1%	1346 out of 28794 genes, 4.7%	3.53E-05
Striated muscle thin filament	9 out of 1129 genes, 0.8%	25 out of 28794 genes, 0.1%	5.28E-05
**Molecular function**			
Hydrolase activity	283 out of 1138 genes, 24.9%	5158 out of 28380 genes, 18.2%	2.08E-06
Cytoskeletal protein binding	101 out of 1138 genes, 8.9%	1398 out of 28380 genes, 4.9%	2.80E-06
Hydrolase activity, acting on acid anhydrides	132 out of 1138 genes, 11.6%	2114 out of 28380 genes, 7.4%	8.62E-05
Pyrophosphatase activity	131 out of 1138 genes, 11.5%	2097 out of 28380 genes, 7.4%	9.39E-05
**Biological process**			
Lucose metabolic process	42 out of 1105 genes, 3.8%	279 out of 27537 genes, 1.0%	2.39E-10
Hexose metabolic process	48 out of 1105 genes, 4.3%	443 out of 27537 genes, 1.6%	7.72E-07
Muscle system process	52 out of 1105 genes, 4.7%	520 out of 27537 genes, 1.9%	2.54E-06
Small molecule metabolic process	215 out of 1105 genes, 19.5%	3657 out of 27537 genes, 13.3%	4.39E-06
Protein activation cascade	11 out of 1105 genes, 1.0%	31 out of 27537 genes, 0.1%	2.49E-05
Monosaccharide metabolic process	48 out of 1105 genes, 4.3%	493 out of 27537 genes, 1.8%	2.50E-05
Muscle contraction	37 out of 1105 genes, 3.3%	328 out of 27537 genes, 1.2%	2.55E-05
Complement activation	9 out of 1105 genes, 0.8%	20 out of 27537 genes, 0.1%	4.39E-05
Alcohol metabolic process	69 out of 1105 genes, 6.2%	854 out of 27537 genes, 3.1%	4.72E-05

KEGG mapping identified a total of 32,664 genes, including 1,504 DEG in 12 categories (P value <0.05) (Additional file 1: Table S3). The complement and coagulation cascade signaling pathways and *Staphylococcus aureus* infection signaling pathway were significantly affected by infection; expression of 60 genes related to the complement and coagulation cascades and 44 genes related to the *Staphylococcus aureus* infection signaling pathway was altered. Other affected immune-related pathways included the phagosome signaling pathway and the antigen processing and presentation signaling pathway, which included 55 and 20 genes, respectively (Table 3).

**Table 3:** **KEGG pathway enrichment analysis of** ***Epinephelus coioides*** **larvae**

List	KEGG pathway	NGS with pathway annotation (1504)	All genes with pathway annotation (32664)	P-value	Q-value	Pathway ID
	**Metabolism**					
	**Carbohydrate metabolism**					
**12**	Glycolysis/Gluconeogenesis	25 (1.66%)	173 (0.53%)	4.10E-07	7.42E-06	ko00010
**18**	Starch and sucrose metabolism	21 (1.4%)	167 (0.51%)	2.98E-05	3.59E-04	ko00500
	**Cellular processes**					
	**Cell communication**					
**9**	Tight junction	112 (7.45%)	1224 (3.75%)	3.60E-12	8.68E-11	ko04530
	**Transport and catabolism**					
**15**	Phagosome	55 (3.66%)	651 (1.99%)	1.27E-05	1.84E-04	ko04145
	**Cell motility**					
**16**	Regulation of actin cytoskeleton	98 (6.52%)	1387 (4.25%)	1.81E-05	2.45E-04	ko04810
	**Organismal systems**					
	**Circulatory system**					
**1**	Cardiac muscle contraction	108 (7.18%)	742 (2.27%)	1.81E-26	3.94E-24	ko04260
**11**	Vascular smooth muscle contraction	88 (5.85%)	988 (3.02%)	2.97E-09	5.86E-08	ko04270
	**Digestive system**					
**2**	Protein digestion and absorption	91 (6.05%)	575 (1.76%)	4.50E-25	4.88E-23	ko04974
**10**	Pancreatic secretion	70 (4.65%)	618 (1.89%)	4.61E-12	1.00E-10	ko04972
	**Immune system**					
**5**	Complement and coagulation cascades	60 (3.99%)	341 (1.04%)	3.13E-19	1.36E-17	ko04610
**17**	Antigen processing and presentation	20 (1.33%)	152 (0.47%)	2.36E-05	3.01E-04	ko04612
	**Diseases**					
	**Infectious diseases: bacterial**					
**6**	Staphylococcus aureus infection	44 (2.93%)	206 (0.63%)	1.00E-17	3.62E-16	ko05150

### Bacterial numbers in *Epinephelus coioides*larvae infected with *V. alginolyticus*

**Figure 2:**
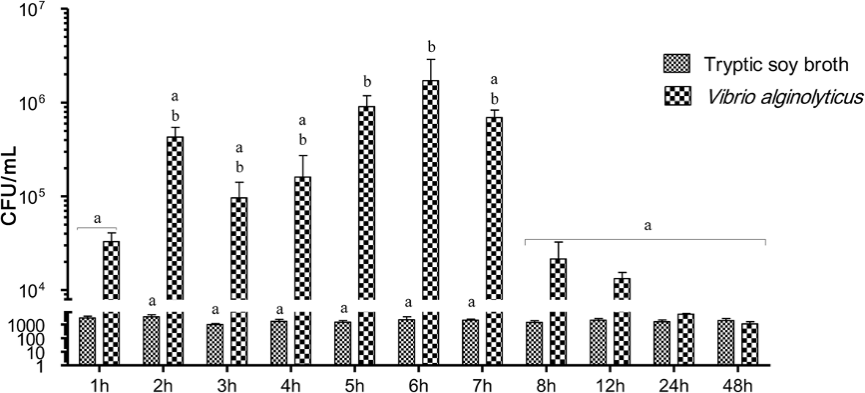
**Colony-Forming Units (CFUs) in** ***Epinephelus coioides*** **larvae infected with** ***V. alginolyticus*** **.** Larvae were injected with 1.3 × 10^6^ CFU/ml *V. alginolyticus* (20 μl per fish). Control fish were injected with Tryptic soy broth containing 1.5% NaCl. Significance was set at P < 0.05, as determined by one-way ANOVA followed by Duncan’s test.

To study *V. alginolyticus* infection in grouper larvae, we calculated the colony-forming units (CFUs) in whole fish over time (Figure 2). CFUs were significantly greater in infected larvae than in control fish between 2 and 7 h post-injection. By 8 h, no significant difference was detected, and by 24 h, CFUs had returned to baseline levels.

### Analysis of immune-related signal transduction pathways in infected fish

GO (Gene Ontology) and KEGG (Kyoto Encyclopedia of Genes and Genomes) analyses of immune factors yielded similar findings; for example, both analyses revealed that complement activation was affected by infection. However, KEGG analysis uncovered additional immune responses and clearly disrupted pathways. We thus subsequently focus on the findings of KEGG analysis. Based on KEGG analysis, we selected the following pathways for analysis: complement and coagulation cascades with a p-value of 3.13 × 10^−19^ (Additional file 2: Figure S1), the *Staphylococcus aureus* infection pathway with a p-value of 1 × 10^−17^ (Additional file 3: Figure S2), the phagosome pathway with a p-value of 1.27 × 10^−5^ (Additional file 4: Figure S3), and the antigen processing and presentation pathway with a p-value of 2.35 × 10^−5^ (Additional file 5: Figure S4). These pathways are part of the teleost immune response. We combined the *Staphylococcus aureus* infection pathway with the complement and coagulation cascades (henceforth referred to as the complement-related pathway), and the phagosome pathway with the antigen processing and presentation pathway (henceforth referred to as the phagocytosis-related pathway), designed primers against unigenes involved in these processes based on the transcriptome sequences, and examined RNA expression by real-time qPCR, as described below.

### Analysis of gene expression in the complement-related pathway

**Figure 3:**
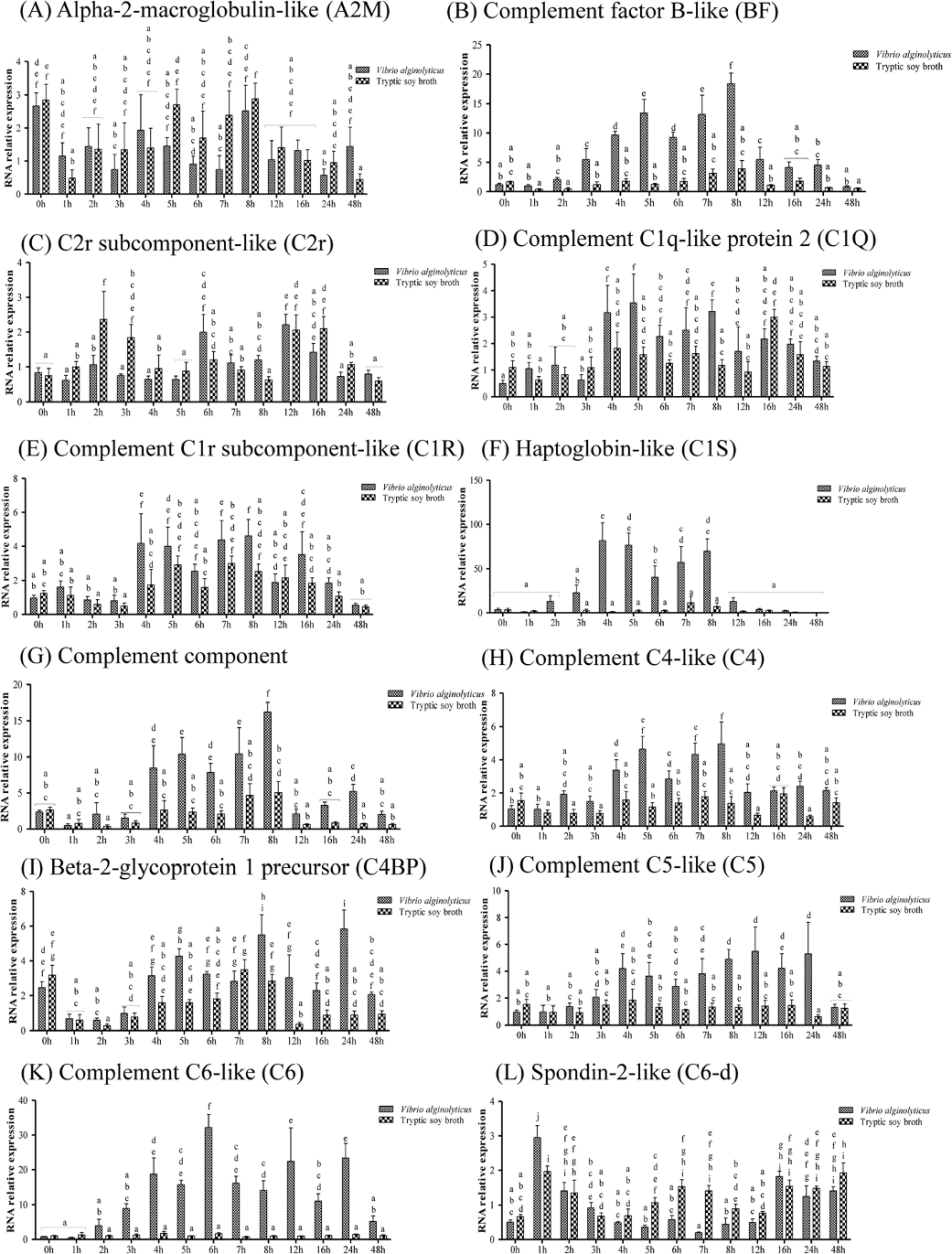
**Complement-related pathway gene expression in controls and grouper challenged with 1.3 × 10** ^**6**^ **CFU/ml (20 μl/fish)** ***Vibrio alginolyticus*** **, as determined by qRT-PCR. (** ***A*** **)** A2M, **(** ***B*** **)** BF, **(** ***C*** **)** CR2, **(** ***D*** **)** C1q, **(** ***E*** **)** C1r, **(** ***F*** **)** C1s, **(** ***G*** **)** C3, **(** ***H*** **)** C4, **(** ***I*** **)** C4BP, **(** ***J*** **)** C5, **(** ***K*** **)** C6, and **(** ***L*** **)** C6-d. Values are presented as the mean ± SEM (n = 5). Values with different letters differ significantly. Significance was set at P < 0.05, as determined by one-way ANOVA followed by Duncan’s test.

**Figure 4:**
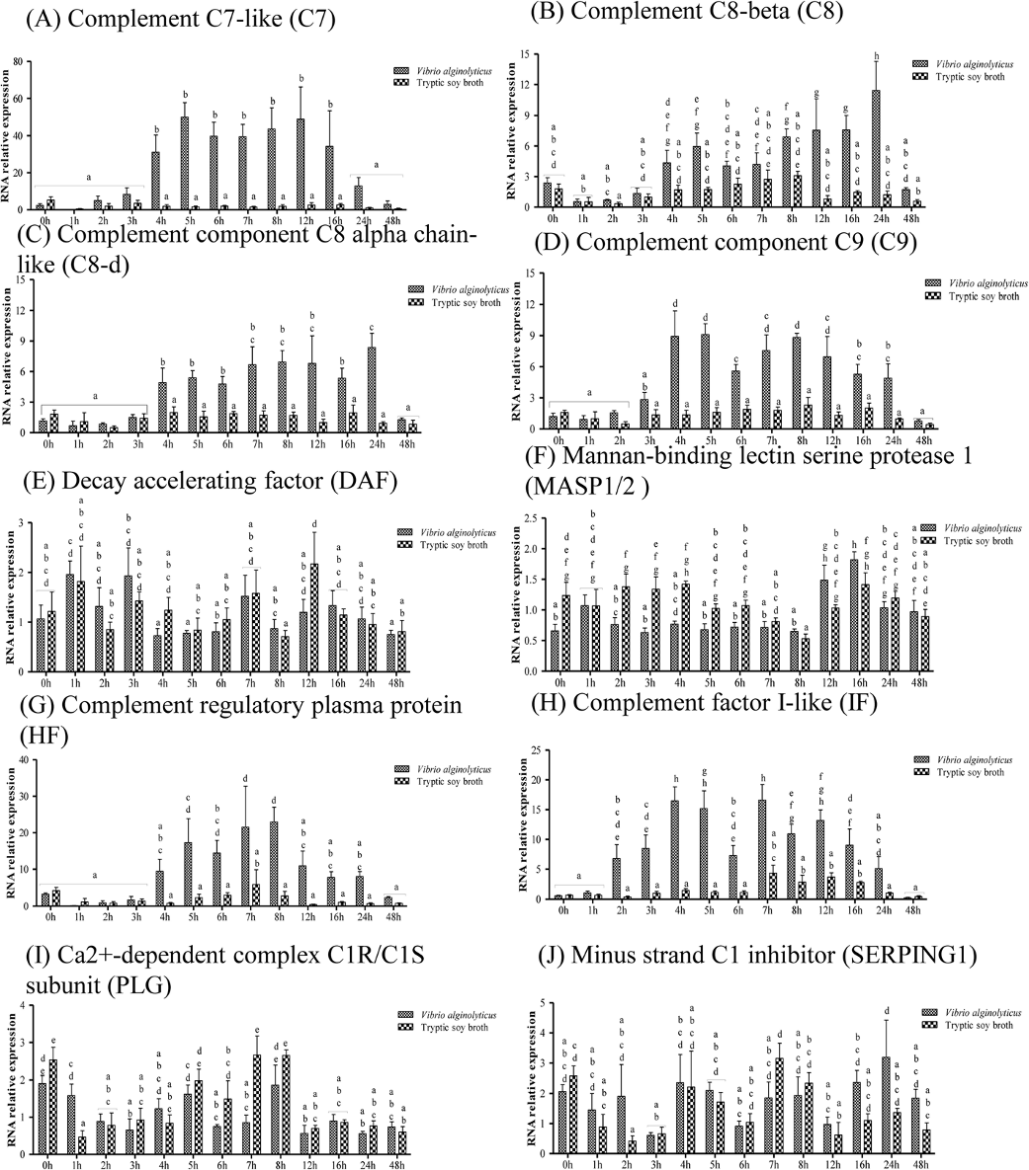
**Complement-related pathway gene expression in controls and grouper challenged with 1.3 × 10** ^**6**^ **CFU/ml (20 μl/fish)** ***Vibrio alginolyticus*** **, as determined by qRT-PCR. (** ***A*** **)** C7, **(** ***B*** **)** C8, **(** ***C*** **)** C8-d, **(** ***D*** **)** C9, **(** ***E*** **)** DAF, **(** ***F*** **)** MASP1/2, **(** ***G*** **)** HF, **(** ***H*** **)** IF **(** ***I*** **)** PLG, and **(** ***J*** **)** SERPING1. Values are presented as the mean ± SEM (n = 5). Values with different letters differ significantly. Significance was set at P < 0.05, as determined by one-way ANOVA followed by Duncan’s test.

As shown in Figures 3 and 4 and Additional file 1: Table S4, we examined the effect of infection on the expression of genes involved in the complement-related pathway by qPCR; the gene names given in the figures are the abbreviations used in KEGG. The complement factor B-like (BF) gene was significantly up-regulated between 3 h and 12 h as compared to the control (Figure 3B), while the C2r subcomponent-like (C2r) gene was significantly down-regulated at 2 h and 3 h (Figure 3C). The complement C1q-like protein 2 (C1q) (Figure 3D) and complement C1r subcomponent-like (C1r) (Figure 3E) genes were significantly elevated in infected larvae at 8 h and 4 h, respectively. Both the haptoglobin-like (C1s) (Figure 3F) and complement component C3 (C3) (Figure 3G) genes were up-regulated between 4 h and 8 h in infected fish. The complement C4-like (C4) gene exhibited an erratic pattern, with up-regulation observed in infected fish at 4 h, 5 h, 7 h, 8 h, and 24 h (Figure 3H). The beta-2-glycoprotein 1 precursor (C4BP) gene was also up-regulated at various time points (5 h, 8 h, 12 h, and 24 h) (Figure 3I). Expression levels of the complement C5 (C5) (Figure 3J) and complement component C6-like (C6) (Figure 3K) genes were significantly increased between 8 h and 24 h, and 4 h to 24 h, respectively. Levels of the Spondin-2-like (C6-d) gene were significantly increased at 1 h, 5 h, 6 h, and 7 h (Figure 3L).The complement component C7-like (C7) gene was significantly elevated between 4 h and 16 h (Figure 4A), and the complement component C8 beta (C8) gene was up-regulated at 5 h, 8 h, 12 h, 16 h, and 24 h in infected fish (Figure 4B). Both the complement component C8 alpha chain-like (C8-d) (Figure 4C) and the complement component C9 (C9) (Figure 4D) genes were significantly increased between 4 h and 24 h. On the other hand, the mannan-binding lectin serine protease 1 (MASP1/2) gene was significantly down-regulated between 2 h and 4 h (Figure 4F). The complement regulatory plasma protein (HF) (Figure 4G) and complement factor I-like (IF) (Figure 4H) genes were significantly up-regulated between 5 h and 8 h, and between 2 h and 16 h, respectively. Finally, the Ca2 + −dependent complex C1r/C1s subunit (PLG) gene exhibited both up- and down-regulation, at 1 h and 7 h, respectively (Figure 4I). Expression levels of alpha-2-macroglobulin-like (A2M) (Figure 3A), decay accelerating factor (DAF) (Figure 4E), and minus strand C1 inhibitor (SERPING1) (Figure 4J) were unaffected by infection. Certain genes were affected in a time-dependent manner, i.e., BF, C1s, C3, C5, C6, C7, C8-d, C9, HF, and IF.

### Analysis of gene expression in the phagocytosis-related pathway

**Figure 5:**
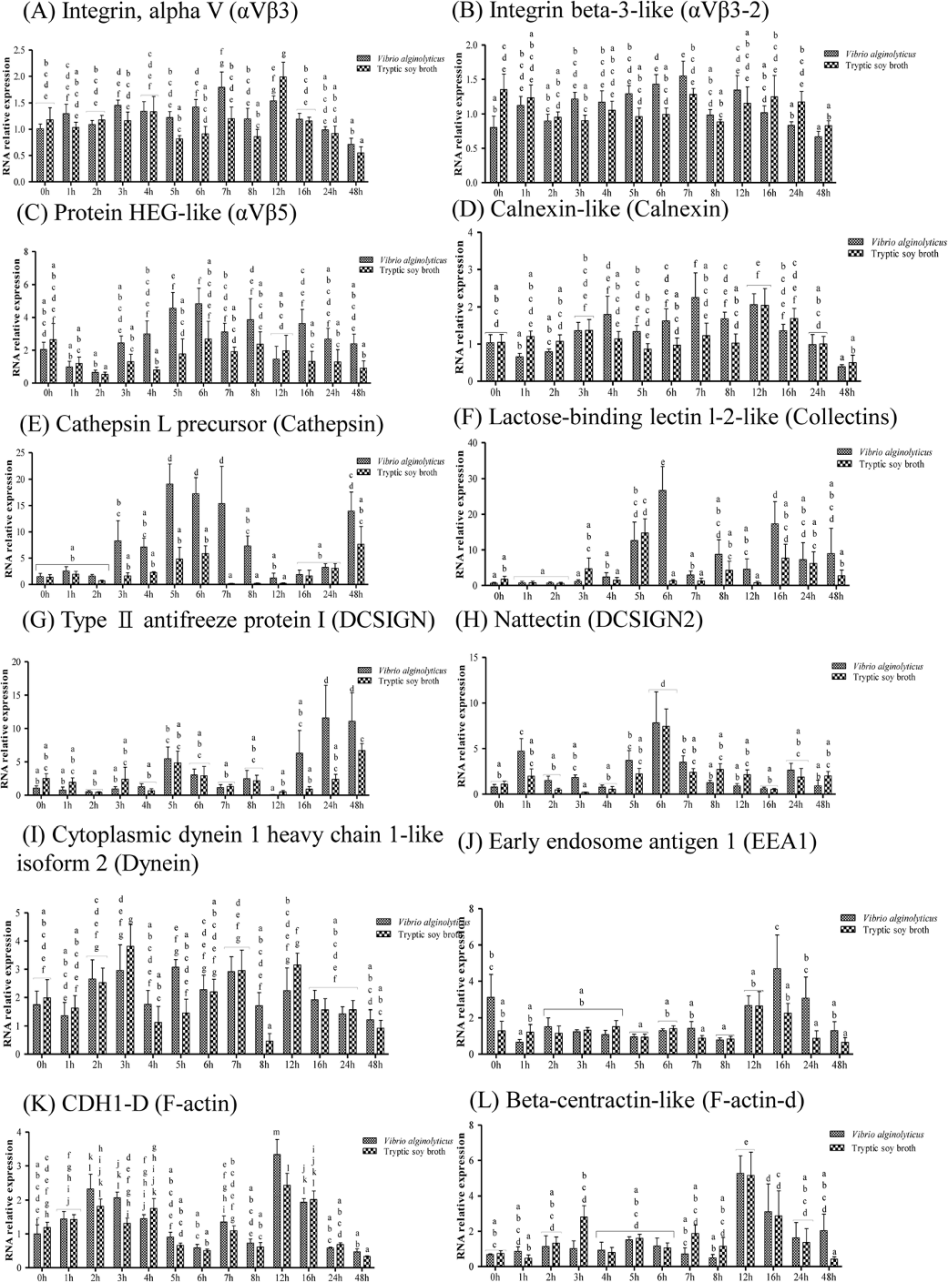
**Phagocytosis-related pathway gene expression in controls and grouper challenged with 1.3 × 10** ^**6**^ **CFU/ml (20 μl/fish)** ***Vibrio alginolyticus*** **, as determined by qRT-PCR. (** ***A*** **)** αVβ3, **(** ***B*** **)** αVβ3-2, **(** ***C*** **)** αVβ5, **(** ***D*** **)** Calnexin, **(** ***E*** **)** Cathepsin, **(** ***F*** **)** Collectins, **(** ***G*** **)** DCSIGN, **(** ***H*** **)** DCSIGN2, **(** ***I*** **)** Dynein, **(** ***J*** **)** EEA1, **(** ***K*** **)** F-actin, and **(** ***L*** **)** F-actin-d. Values with different letters differ significantly. Values are presented as the mean ± SEM (n = 5). Significance was set at P < 0.05, as determined by one-way ANOVA followed by Duncan’s test.

**Figure 6:**
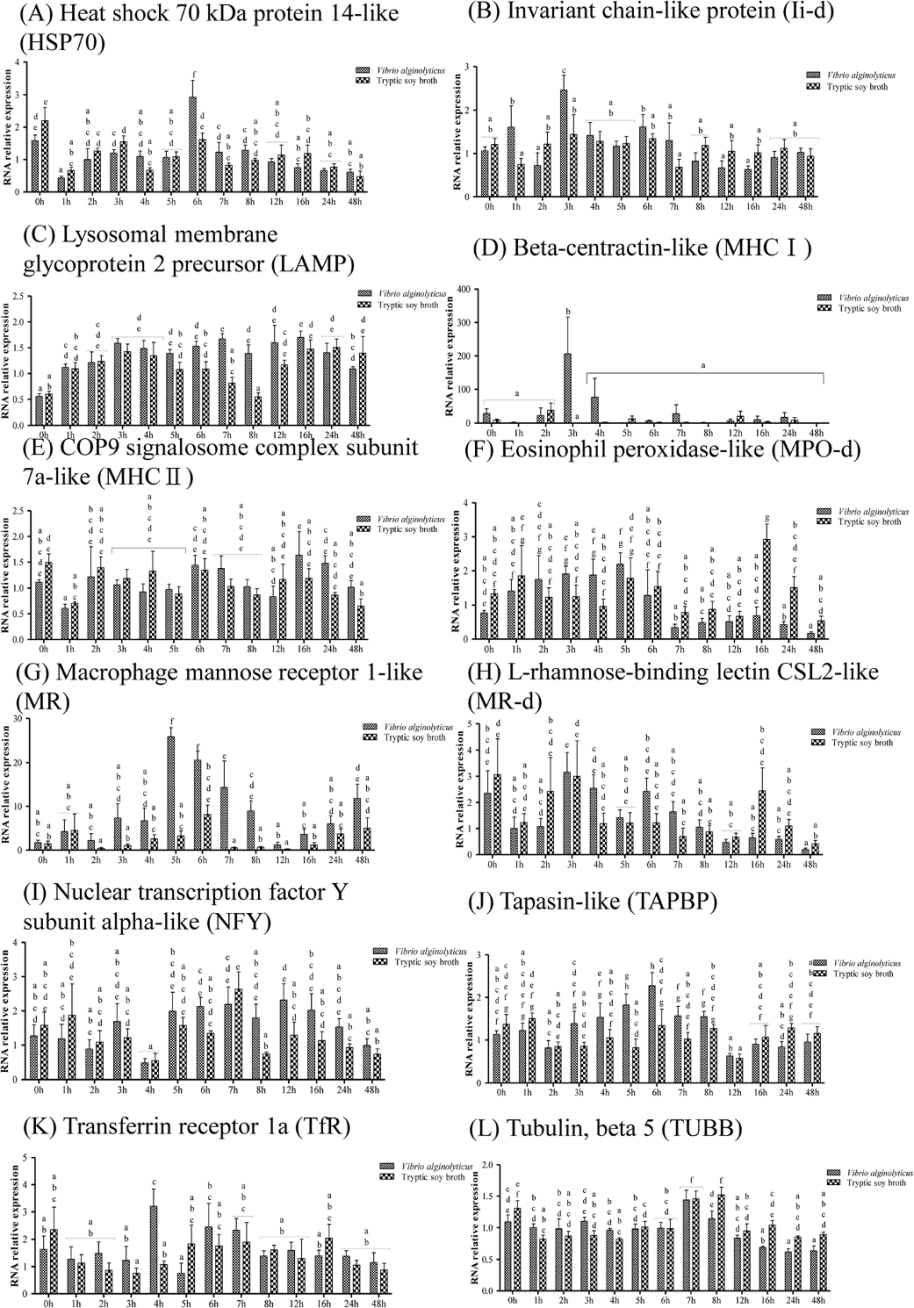
**Phagocytosis-related gene expression in controls and grouper challenged with 1.3 × 10** ^**6**^ **CFU/ml (20 μl/fish)** ***Vibrio alginolyticus*** **, as determined by qRT-PCR. (** ***A*** **)** HSP70, **(** ***B*** **)** Ii-d, **(** ***C*** **)** LAMP, **(** ***D*** **)** MHCI, **(** ***E*** **)** MHCII, **(** ***F*** **)** MPO-d, **(** ***G*** **)** MR, **(** ***H*** **)** MR-d, **(** ***I*** **)** NFY, **(** ***J*** **)** TAPBP, **(** ***K*** **)** TfR, and **(** ***L*** **)** TUBB Values are presented as the mean ± SEM (n = 5). Values with different letters differ significantly. Significance was set at P < 0.05, as determined by one-way ANOVA followed by Duncan’s test.

The effect of infection on the expression of genes in the phagocytosis-related pathway was more erratic than that on genes of the complement-related pathway (Additional file 1: Table S5). The protein HEG-like (αVβ5) (Figure 5C) and Calnexin-like (Calnexin) (Figure 5D) genes were significantly up-regulated by infection at 5 h and 7 h, respectively. Cathepsin L precursor (Cathepsin) gene was up-regulated between 5 h and 7 h (Figure 5E), while the lactose-binding lectin l-2-like (Collectins) gene was up-regulated at 6 h only (Figure 4F). Expression of the type II antifreeze protein I (DCSIGN) gene was elevated at 24 h and 48 h (Figure 5G), that of the early endosome antigen 1 (EEA1) gene at 16 h and 24 h (Figure 5J), and that of the CDH1-D (F-actin) gene at 12 h (Figure 5K).The heat shock 70 kDa protein 14-like (HSP70) gene was significantly up-regulated by infection at 6 h (Figure 6A), and the invariant chain-like protein (Ii-d) gene at 3 h (Figure 6B). The lysosomal membrane glycoprotein 2 precursor (LAMP) gene was up-regulated at both 7 h and 8 h (Figure 6C). The beta-centractin-like (MHCI) gene was also up-regulated at 3 h (Figure 6D), while the eosinophil peroxidase-like (MPO-d) gene was down-regulated at 16 h (Figure 6F). Infection increased expression of the macrophage mannose receptor 1-like (MR) gene between 5 h and 8 h (Figure 6G), the tapasin-like (TAPBP) gene at 5 h and 6 h (Figure 6J), and the transferrin receptor 1a (TfR) gene at 4 h (Figure 6K). Finally, the tubulin, beta 5 (TUBB) gene was significantly down-regulated at 8 h and 16 h (Figure 6L). Infection did not affect the expression levels of Integrin, alpha V (αVβ3) (Figure 5A), Integrin beta-3-like (αVβ3-2) (Figure 5B), Nattectin (DCSIGN2) (Figure 5H), Cytoplasmic dynein 1 heavy chain 1-like isoform 2 (Dynein) (Figure 5I), Beta-centractin-like (F-actin-d) (Figure 5L), COP9 signalosome complex subunit 7a-like (MHCII) (Figure 6E), L-rhamnose-binding lectin CSL2-like (MR-d) (Figure 6H), or Nuclear transcription factor Y subunit alpha-like (NFY) genes (Figure 6I).

### Analysis of antimicrobial peptide gene expression

The innate immune response includes antimicrobial peptides, which can damage bacterial membranes. We identified three antimicrobial peptide genes in the transcriptome library, and analyzed their expression in infected larvae over time (Additional file 1: Table S6). Interestingly, expression of the antimicrobial peptide Epinecidin-1, which our laboratory previously isolated from *Epinephelus coioides*[9], was unaffected (data not shown). The sequence of epinecidin-1 is similar to that of piscidin (54% identity), and these two peptides were found to have similar effects. However, here we focused on the three antimicrobial peptide genes in our transcriptome database.

**Figure 7:**
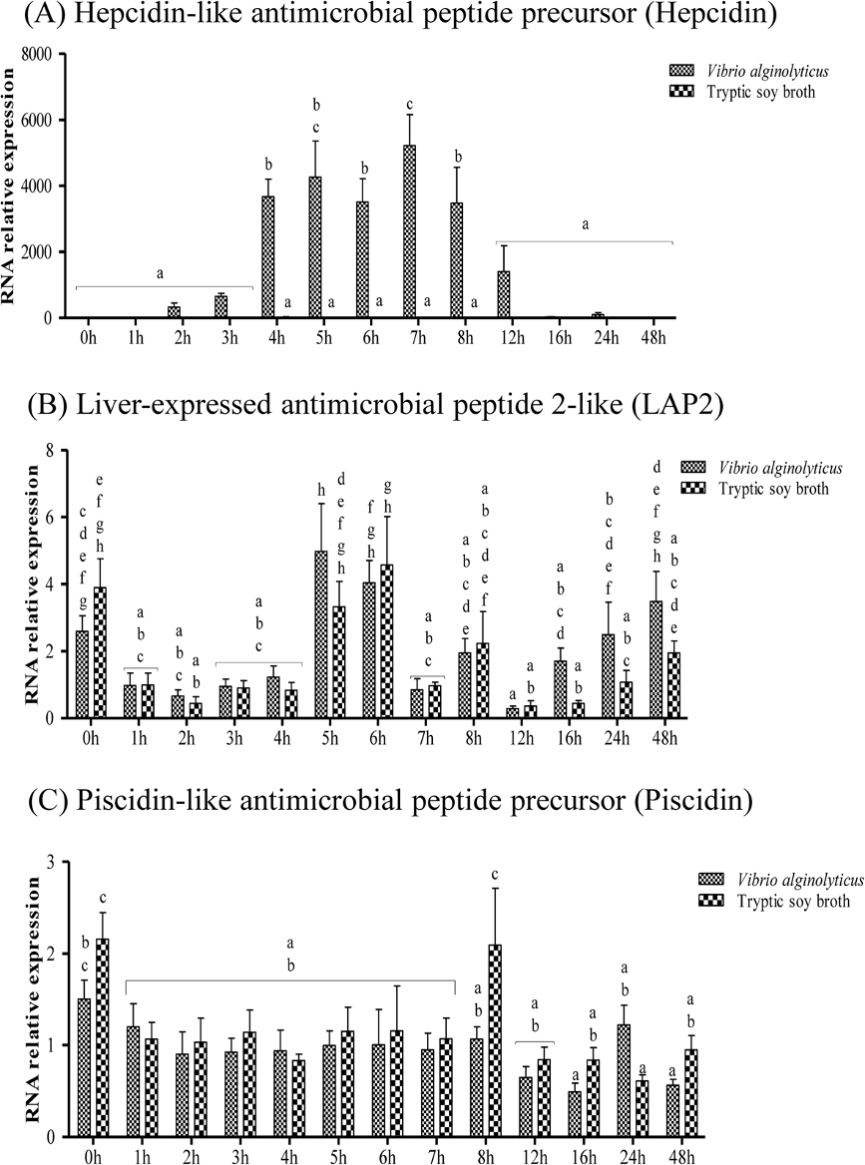
**Antimicrobial peptide gene expression in control and grouper challenged with 1.3 × 10** ^**6**^ **CFU/ml (20 μl/fish)** ***Vibrio alginolyticus*** **, as determined by qRT-PCR. (** ***A*** **)** Hepcidin, **(** ***B*** **)** LAP2, and **(** ***C*** **)** Piscidin. Values are presented as the mean ± SEM (n = 5). Values with different letters differ significantly. Significance was set at P < 0.05, as determined by one-way ANOVA followed by Duncan’s test.

The hepcidin-like antimicrobial peptide precursor (Hepcidin) gene was significantly up-regulated by infection from 4 h to 8 h (Figure 7A), while the liver-expressed antimicrobial peptide 2-like (LAP2) was unaffected (Figure 7B). Conversely, the Piscidin-like antimicrobial peptide precursor (Piscidin) gene was significantly down-regulated at 8 h (Figure 7C). These findings suggest that *Epinephelus coioides* is dependent on innate immunity to defend against *V. alginolyticus* infection.

### Analysis of the complement- and phagocytosis-related pathways by KEGG

As described above, we combined the complement and coagulation cascades with the *Staphylococcus aureus* infection pathway to form the complement-related pathway (Additional file 6: Figure S5A), and the phagosome pathway with the antigen processing and presentation pathway to form the phagocytosis-related pathway (Additional file 6: Figure S5B). The complement-related pathway transduces signals to classical pathways, the alternative pathway, and lectin pathway. All complement-related pathways involve cleavage of C3 to C3b and then to C5, in order to form a membrane attack complex (MAC) for bacterial lysis. The phagocytosis pathway uses nitric oxide synthase to produce NO via the phagosome, which then digests the bacterium and presents the antigen fragment, thereby activating MHCI and MHCII to stimulate the adaptive immune system.

## Discussion

Here, we describe the use of NGS technology to uncover the response of the transcriptome of *Epinephelus coioides* larvae to infection by *V. alginolyticus*. Few studies have focused on infection of the grouper larvae stage; however, because the long dorsal fin of the larvae stage is yet to completely disappear, it makes fish of this developmental stage prone to getting trapped in nets. Furthermore, they are susceptible to dying for many reasons, including changes in water temperature, aeration rate, salinity, and illumination [10]. We constructed a transcriptome library from *Epinephelus coioides* larvae, as transcriptome profiling is a powerful method for evaluating the relative importance of gene products in a given tissue [11], and it enabled us to determine the effects of infection on gene expression at the larval stage. We deduced the immune-related signal transduction pathway from KEGG enrichment analysis. This pathway was predicted to consist of the complement and coagulation cascades, *Staphylococcus aureus* infection pathway, phagosome pathway, and antigen processing and presentation pathway. The complement system is an ancient mechanism, found in both protostome and invertebrate deuterostome species [12–14]. Like other higher vertebrates, teleost fish contain three complement pathways. Earlier studies have demonstrated that the alternative and classical pathways have a significant effect in teleost fish [15]. However, very little is known about the molecules involved in the lectin pathway in fish [16]. The complement components of fish are different to those of mammals, and some consist of multiple isoforms [17–19]. The complement pathway is known to be one of the key mechanisms for bacterial clearance in teleost fish [20, 21]; the alternative pathway can be activated by the lipopolysaccharides (LPS) of Gram-negative bacteria, enabling lysis of the bacterial cell [22]. Here, we observed that infection had time-dependent effects on several genes related to the complement pathway; however, of the genes of the phagocytosis-related pathway, only the MR and cathepsin genes were affected in a time-dependent manner. The complement factor B-like (BF) gene cleaves C3 and acts as a convertase in rainbow trout [23]. Although the C1r/C1s/MASP-like genes of grouper have not been functionally characterized, previous findings suggest that the C1s-like molecule may cleave C4 to C4a and C4b fragments in rainbow trout [15, 24].

In teleost fish, C4 plays an important role in activation of the classical pathways [15]. However, our current results indicate that C4 gene expression is not affected in a time-dependent manner by *V. alginolyticus* infection in grouper larvae. Mammalian C3 is encoded by a single gene, but almost all teleost fish studied produce multiple forms of C3 encoded by different genes [16]. In trout, carp, and seabream, these C3 isoforms exhibit different binding efficiencies to several active complements; as such, these isoforms may perform separate roles in the destruction of microbes and innate recognition [25]. In *Epinephelus coioides*, C3 may be inducible and involved in stress responses [3]. C5 is a part of the membrane attack complex (MAC), which cleaves C5 into C5a and C5b fragments [26]. The C5 gene has been partially cloned and purified from trout and seabream [27, 28], and MAC has been shown to consist of C5b, C6, C7, and the beta chains of C8 and C9 in these species [29, 30]. It has been hypothesized that complement cofactor protein (SBP1) regulates both C4b binding protein and factor H [31] in barred sand bass (*Paralabrax nebulifer*), and two factor I isotypes have been identified in carp [32]. Based on our results, we hypothesize that *V. alginolyticus* infection of orange-spotted grouper initially results in the activation of genes such as BF and IF, which mediate C3 production between 2 and 3 h post-infection. C3 is then cleaved by C3 convertase through the alternative or classical pathway at 4 h (via C1s or C4). Formation of MAC occurs between 4 and 5 h (although C5 expression is not significantly up-regulated by infection until 8 h, a non-significant increase can be observed at 4 h). Finally, MAC may clear *V. alginolyticus* before 48 h.

**Figure 8:**
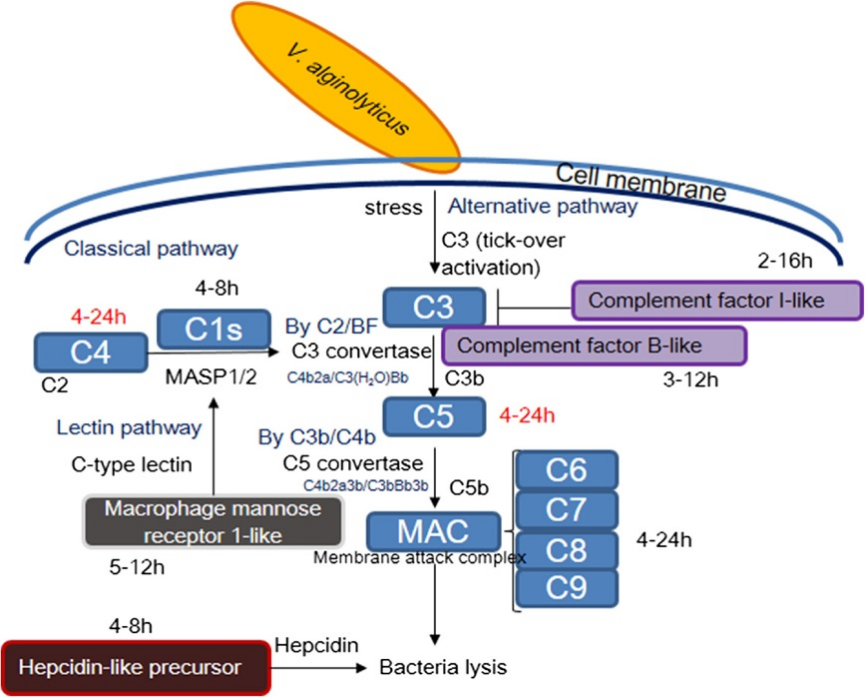
**Predicted model of the immune response of** ***Epinephelus coioides*** **larvae to** ***V. alginolyticus*** **infection.** The times (in h) besides each gene indicate the time post-*V. alginolyticus* infection at which its expression is significantly increased (red font indicates uncertainty). The classical pathway involves (i) cleavage of C4 to C3 convertase by C1s, (ii) cleavage of C3 to C3b by C3 convertase, (iii) combination of C3b with C5, (iv) cleavage of the resulting complex to C5b by C5 convertase, and (v) formation of the membrane attack complex (MAC) and lysis of *V. alginolyticus*. The lectin pathway involves C-type lectin-mediated cleavage of C4 and C2 by MASP1/2, and subsequent cleavage of C3 by C3 convertase. The alternative pathway involves cleavage of C3 by factor B; like the classical pathway, factor I acts as an inhibitor in the alternative pathway. Hepcidin may directly kill the bacterium by disrupting its membrane.

The phagosome and antigen processing system include phagocytic cells (granulocytes, monocytes, and macrophages), non-specific cytotoxic cells, and dendritic cells [33, 34]. From our gene expression results, it is difficult to determine how infection affects the phagocytosis-related pathway (Figure 8). However, we did observe an increase in macrophage mannose receptor (MR) expression between 5 and 8 h, and thus conclude it may be involved in the complement lectin pathway and initiated by the binding of a lectin, such as C-type lectin; it may then activate the complement pathway upon binding of a collectin to a microbial surface [35]. The MR may also be involved in the phagocytosis of yeast cells by head-kidney leucocytes in seabream (*Sparus aurata L*.) [36]. Complement component C3 has been identified in fertilized cod eggs [37], and phagocytic activity has been detected in zebrafish embryo and 2-day-post-fertilization carp embryo [38, 39].

Antimicrobial peptides play important roles in the innate immune response to bacterial infection. Antimicrobial peptides range in size from 6 amino acid residues for anionic peptides, to as many as 59 amino acid residues; larger proteins possess several features of secondary structure, including α -helices, relaxed coils, and antiparallel β-sheet structures. Such features are hydrophobic, which enables water-soluble antimicrobial peptides to pass through the membrane lipid bilayer. Antimicrobial peptides kill bacteria via one of three mechanisms, known as the barrel-stave model, carpet model, and toroidal model. The barrel-stave model involves aggregation of peptides at the membrane bilayer; the hydrophobic peptide regions align to form a lipid hole like a barrel, disrupting the osmotic balance of the bacterial inner membrane. In the carpet model, the peptides are oriented parallel to the surface of the lipid bilayer, like a carpet. In the toroidal model, the peptides aggregate, and form a pore in the lipid monolayers, and the inserted peptides and the lipid head groups induce a water core line [40].

Hepcidin was first identified as a protein involved in innate immunity and iron regulation in the human liver. Teleost fish hepcidin has been previously demonstrated to be involved in both iron regulation and immunity. Seabream hepcidin is abundant in liver, skin, head-kidney, and peritoneal exudate leucocytes, and flounder hepcidin-like is distributed in liver, esophagus, and cardiac stomach. Gene expression is up-regulated by poly I:C, iron dextran, bacteria, or LPS [41, 42]. In a recent study, a four-cysteine hepcidin isoform gene, EC-hepcidin3, was cloned from *Epinephelus coioides*, and was reported to be effective against *Staphylococcus aureus* and *Pseudomonas stutzeri*[43]. Human hepcidin is induced by IL-6 [44], and we previously observed that transgenic zebrafish expressing tilapia hepcidin 2–3 had higher transcript levels of IL-10, IL-26, TLR4a, and TNF-α as compared with wild-type zebrafish [45]. The gene encoding the hepcidin-like antimicrobial peptide precursor was strongly induced by infection at 7 and 8 h, and signs of increased expression suggest that grouper larvae may be dependent on hepcidin function from 4 to 8 h.

## Conclusions

In conclusion, the present study suggests that the response of *Epinephelus coioides* larvae to *V. alginolyticus* infection is dependent on the complement pathway and antimicrobial peptides. Hepcidin, which plays important roles during the larvae stages of grouper, may also be involved in the defense against bacterial infection (Figure 8). These results may be useful to research on fish, as they suggest that the complement pathway and antimicrobial peptides may be beneficial in terms of enhancing grouper anti-bacterial defenses.

## Methods

### Fish and bacteria

*Epinephelus coioides* larvae were purchased from the Institute of Biotechnology, National Cheng Kung University Core Facility. The *Epinephelus coioides* larvae were kept in a 2 tonne FRP tank prior to bacterial infection. *Vibrio alginolyticus* were cultured as previously described [46]. The animal experimental and ethics protocol (12-12-447) was approved by the Academia Sinica Institutional Animal Care and Use Committee (IACUC) of the Institute of Cellular and Organismic Biology, Academia Sinica, Taiwan.

### Bacterial infection and colony-forming unit (CFU) counts

At 30-days-old, the whole bodies of *Epinephelus coioides* larvae with an average body length of about 1.6 ± 0.2 cm were injected with 20 μl (1.3 × 10^6^ colony-forming units (CFU)/ml) of *V. alginolyticus* in Tryptic soy broth (NaCl 1.5%). *Vibrio* were detected by spreading the culture onto thiosulfate–citrate–bile salt–sucrose agar (TCBS) plates, and incubating the plates at 28°C for 16 h; *V. alginolyticus* forms yellow colonies [46]. Control grouper were injected with TSB (NaCl 1.5%) without *V. alginolyticus*. Fish were sacrificed at 1, 2, 3, 4, 5, 6, 7, 8, 12, 24, and 48 h after infection (five fish were sacrificed at each time point for each group). Whole fish were homogenized using Lyser II (Qiagen, USA) solution, and CFU were determined as previously described [47].

### Epinephelus coioides larval RNA preparation for next generation sequencing

*Epinephelus coioides* larvae (30-days-old) were injected with *V. alginolyticus* as described in the preceding section, and fish were sacrificed at 24 h after infection. Ten wild-type *Epinephelus coioides* larvae were used to build an EST library for transcriptome analysis. RNA was extracted as previously described [48]. RNA concentrations were quantified using a Nano-Drop spectrophotometer (Thermo, USA), and quality was determined with an RNA gel, as described in *Molecular Cloning*[44]. RNA samples for real-time qPCR were extracted from fish sacrificed at 1, 2, 3, 4, 5, 6, 7, 8, 12, 24, and 48 h after infection (n = 5); three independent trials were performed.

Oligo (dT) magnetic beads were used to enrich mRNA, which were then broken into short fragments (about 200 bp) in fragmentation buffer; these fragments were then reverse transcribed into first strand cDNA using random hexamer primers. The appropriate buffer, dNTPs, RNase H, and DNA polymerase I were added to synthesize second-strand cDNA. Double-stranded cDNA was purified with the QiaQuick PCR extraction kit, and washed with EB buffer. Sequencing adaptors were ligated to the fragments. The required fragments were purified by agarose gel electrophoresis and copied by PCR amplification. The library products were prepared for sequencing analysis using an Illumina HiSeq™ 2000. Raw data were saved as fastq files. The following were removed: reads with adaptors, reads in which over 10% bases were unknown, and low quality reads (i.e., the percentage of bases of quality value ≤5 exceeds 50% in the read). Clean reads were mapped to reference sequences using SOAP aligner/soap2 [49]. The randomness of RNA fragmentation was used to construct the library, and the numbers of reads mapped to the reference sequence were calculated. The RPKM method (Reads Per kb per Million reads) was used to calculate gene expression level [50], and differentially expressed genes (DEGs) were subsequently screened for as previously described [51]. DEGs were subjected to GO function and KEGG pathway analysis, as described below. All transcriptome databases used in our study can be downloaded from the foot of GSE63148 used by NCBI Gene Expression Omnibus (http://www.ncbi.nlm.nih.gov/geo/query/acc.cgi?acc=GSE63148); this includes bacteria-infected group gene rpkm (GSE63148_B4_1.Gene.rpkm.txt.gz), control group gene rpkm (GSE63148_MRS17.Gene.rpkm.txt.gz), transcriptome EST library (GSE63148_MRS-16-Unigene.fa.gz), gene differential expression (GSE63148_MRS17-VS-B4_1.GeneDiffExp.txt.gz), and gene annotation (GSE63148_annotation.txt.gz).

### Gene ontology (GO) and Kyoto encyclopedia of genes and genomes (KEGG) analysis

GO enrichment analysis was performed by collating all the GO terms that were significantly enriched in the identified DEG, and then filtering the DEGs based on these biological functions. First, all DEGs were mapped to GO terms in the database (http://www.geneontology.org/), and then gene numbers were calculated for every term using the hypergeometric test in order to obtain significantly enriched GO terms for DEGs; these were compared to the genomic background, as described in a previous study [52]. Pathway enrichment analysis was performed using KEGG (http://www.genome.jp/kegg/), which is a public pathway-related database. Such analysis was used to identify significant enrichment of genes involved in metabolic or signal transduction pathways. DEGs were compared with the genomic background, and the formula was calculated as for GO analysis.

### Analysis of gene expression

The real-time PCR primers used in this study are described in Additional file 1: Table S1; real-time qPCR was performed as previously described [46].

## Electronic supplementary material

Additional file 1: Table S1.: Real-Time PCR primers used in this study. **Table S2.** Gene ontology analysis of *Epinephelus coioides* larvae. **Table S3.** KEGG pathway enrichment analysis of *Epinephelus coioides* larvae. **Table S4.** Summary of the variations in complement-related gene expression between controls and grouper challenged with *Vibrio alginolyticus*. **Table S5.** Summary of the variations in phagocytosis-related gene expression between controls and grouper challenged with *Vibrio alginolyticus*. **Table S6.** Summary of the variations in antimicrobial peptide gene expression between controls and grouper challenged with *Vibrio alginolyticus*. (PDF 360 KB)

Additional file 2: Figure S1.: Complement and coagulation cascades signal pathway. Enrichment analysis of DEGs from the KEGG database; red borders indicate up-regulated genes, green borders indicate down-regulated genes, and red/green borders indicate genes that are both up- and down-regulated at different times. (TIFF 233 KB)

Additional file 3: Figure S2.: *Staphylococcus aureus* infection signal pathway. Enrichment analysis of DEGs from the KEGG database; red borders indicate up-regulated genes, green borders indicate down-regulated genes, and red/green borders indicate genes that are both up- and down-regulated at different times. (TIFF 280 KB)

Additional file 4: Figure S3.: Phagosome signal pathway. Enrichment analysis of DEGs from the KEGG database; red borders indicate up-regulated genes, green borders indicate down-regulated genes, and red/green borders indicate genes that are both up- and down-regulated at different times. (TIFF 259 KB)

Additional file 5: Figure S4.: Antigen processing and presentation signal pathway. Enrichment analysis of DEGs from the KEGG database; red borders indicate up-regulated genes, green borders indicate down-regulated genes, and red/green borders indicate genes that are both up- and down-regulated at different times. (TIFF 177 KB)

Additional file 6: Figure S5.: Hypothetical model for the response of grouper larvae to *Vibrio alginolyticus* infection, as predicted by KEGG analysis. (A) Complement pathway. (B) Phagocytosis pathway. Red borders indicate up-regulated genes, green borders indicate down-regulated genes, and purple borders indicate genes that are both up- and down-regulated at different times. Red arrows indicate increased RNA expression and green arrows indicate decreased RNA expression. Numbers adjacent to borders are the log2 ratio of significantly affected genes. (TIFF 287 KB)
