# Supplementary material for: Transcriptome analysis of the effect of Vibrio alginolyticus infection on the innate immunity-related complement pathway in Epinephelus coioides
Source: BMC Genomics. 2014 Dec 13;15(1):1102. doi: 10.1186/1471-2164-15-1102 (PMC4407539; doi:10.1186/1471-2164-15-1102)
Supplement: Supplementary file 1 — Additional file 1: Table S1.: Real-Time PCR primers used in this study. Table S2. Gene ontology analysis of Epinephelus coioides larvae. Table S3. KEGG pathway enrichment analysis of Epinephelus coioides larvae. Table S4. Summary of the variations in complement-related gene expression between controls and grouper challenged with Vibrio alginolyticus. Table S5. Summary of the variations in phagocytosis-related gene expression between controls and grouper challenged with Vibrio alginolyticus. Table S6. Summary of the variations in antimicrobial peptide gene expression between controls and grouper challenged with Vibrio alginolyticus. (PDF 360 KB) [file 12864_2014_6984_MOESM1_ESM.pdf]

Supplemental Table 1: Real-Time PCR primers used in this study

Genes	Predicted	Sequences	Species
αVβ3	Integrin, alpha V	F: GTTGGACCGTCTGAAGC	*Danio rerio*
		R: CCGAAACTCACGGTCATC	
αVβ3-2	integrin beta-3-like	F: GGAGTCCCAGAAGAATTGAAC	*Oreochromis niloticus*
		R: AAGTACGGGTCTTCTCTTTG	
αVβ5	protein HEG-like	F: AGATTGTCCGTTCGGTTAT	*Oreochromis niloticus*
		R: TGTCCTTCTTGGAGCTCT	
A2M	alpha-2-macroglobulin-like	F：ATGAGGACAACTGTGTTGA	*Oreochromis niloticus*
		R：GAGGGACACATCCTTCG	
β-actin	β-actin	F：GAGAGGGAAATCGTGCG	*Epinephelus coioides*
		R：ACGGAACCTCTCATTGC	
BF	complement factor B-like	F：GTCAAGAGAAGAATATCTGGACATTTA	*Oreochromis niloticus*
		R：GACCACACAGACCCTTAAC	
Calnexin	calnexin-like	F: TGTGGAGAAGATTACAAACTGC	*Oreochromis niloticus*
		R: GTTCACCACCAGTGTGTAGA	
Cathepsin	Cathepsin L precursor	F: TGGAAGGTCCTACAACTCTC	*Anoplopoma fimbria*
		R: GCGTCTGTACTCTTCGTTT	
Collectins	lactose-binding lectin l-2-like	F: GTCCGATGGGTCTAGAGT	*Oreochromis niloticus*
		R: GGGATACACAAACAGAAGCG	
C2r	C2r subcomponent-like	F：GAACCGTCCGTCCAAAC	*Danio rerio*
		R：GGTCTGATTATCCTGGCAGAA	

C1q	Complement C1q-like protein 2	F：ACTGATGCAGGATATATTGGAC	*Salmo salar*
		R：ACACACAGTGAACTGGAG	
C1r	complement C1r subcomponent-like	F：GGTTTAATATCACTCTCAACTTTAGCGA	*Oreochromis niloticus*
		R：AGCTATCAGACCTGGACT	
C1s	haptoglobin-like	F：GGCAGAAACCTGTTTGTCA	*Oreochromis niloticus*
		R：GTAGTTCTGGAAGTGTGGATG	
C3	complement component C3	F：CCTCAACAAGTTTGCTTCC	*Epinephelus coioides*
		R：TTATAGTAGCCTGAGTTGATCCGTA	
C4	complement C4-like	F：CTGATTCTATCACCACATGGG	*Oreochromis niloticus*
		R：TAACAGGTGAAATGGATAGTTGC	
C4BP	Beta-2-glycoprotein 1 precursor	F：CCCTGACCCAGTGTCTAA	*Anoplopoma fimbria*
		R：CACAGGACACAGGTATGC	
C5	complement C5	F：CTGGAGAAGAAGCTGCG	*Oreochromis niloticus*
		R：AGCTGTGTTTGGAACGA	
C6	complement component C6-like	F：ACAATGATGATGACTACACTATAGG	*Oreochromis niloticus*
		R：CAGTGTAGCACTGGAACTC	
C6-d	spondin-2-like	F：GGCCAGACGAATACTGAG	*Oreochromis niloticus*
		R：TGTCAGTTCCTGCGTCAT	
C7	complement component C7-like	F：CGTTTCTTGAGGAGGAAAGT	*Oreochromis niloticus*
		R：GAGGCCCAGTTGTCGTA	
C8	complement component C8 beta	F：CCTTGCCACAGTCCTAT	*Paralichthys olivaceus*
		R：CGTAGTCGTCCAAAGTGTAATC	

C8-d	complement component C8 alpha chain-like	F：ACTACCATACCTACCAGTTTG	*Oreochromis niloticus*
		R：TCAGTCCCACTTCCACATAAT	
C9	complement component C9	F：AGGGAAATGCTGAAAGAGAT	*Oreochromis niloticus*
		R：GTACTCTGAGACCTCACGA	
DAF	Decay accelerating factor	F：ATTGACATGGACAGAGGAAG	*Danio rerio*
		R：ATGGCTGTTTACCTGTGG	
DCSIGN	type II antifreeze protein I	F: GGTGTTTGGCTCTGGAA	*Epinephelus bruneus*
		R: TCTTGGCGCAGACAGAA	
DCSIGN2	nattectin	F: GCCTTCATTTCATTGCGG	*Epinephelus bruneus*
		R: TCCTTTGAAGCATGGTGGTA	
Dynein	cytoplasmic dynein 1 heavy chain 1-like isoform 2	F: GACAGACCGCTGCTATC	*Oreochromis niloticus*
		R: ATGGCCTGGAAGTCGAA	
EEA1	early endosome antigen 1	F: GGAGGATGAATCTGAACGC	*Oreochromis niloticus*
		R: GCTTTGCTGCTGCAAAT	
F-actin	CDH1-D	F：AGTCAAGTTTGATCGTCTTCTC	*Gallus gallus*
		R：AGTAAGCGCGGGTCATA	
F-actin-d	beta-centractin-like	F：CTGGTGTTGTGTTGGACT	*Oreochromis niloticus*
		R：TGTTGAAATTGTAGCCTTCCT	
Hepcidin	Hepcidin-like antimicrobial peptide precursor	F: GCTCAGTGTTGCAGTTG	*Epinephelus coioides*
		R: CTCGATATTAAATGGCATCATCC	
HF	complement regulatory plasma protein	F：ATGGAATCCAGATCCACTTT	*Paralabrax nebulifer*
		R：TCTGTGTTTCCGTATTCCCT	

HSP70	heat shock 70 kDa protein 14-like	F：CCGAATTCAAACGCACC<br>R：TCTGGATATGTTGCATTCAAAGT	*Oreochromis niloticus*
IF	complement factor I-like	F：GGATGTGGTGAGGAGTTG<br>R：ACTCACACATTTGGTGGGTAA	*Oreochromis niloticus*
Ii	invariant chain-like protein	F: GAGCTTTGAGTCCTGGAT<br>R: GGCTTGTAGGAACCGAT	*Epinephelus akaara*
LAMP	lysosomal membrane glycoprotein 2 precursor	F: ATTCTTCCCTAAAGCATCAACAA<br>R: GCCTGTATGAGCACGTTC	*Salmo salar*
LAP2	Liver-expressed antimicrobial peptide 2-like	F: AGGTCAATTCACTGCCTGTA<br>R: GCAGATATTCTTCCTGCAGTA	*Oreochromis niloticus*
MASP1/2	Mannan-binding lectin serine protease 1	F：ACAAAGGACAAGAAGAGAAGG<br>R：GTTCTTTGTCATTTCCAGTTGAAATA	*Oreochromis niloticus*
MHCI	MHC class I alpha antigen	F：TTGAGATTGTAAAGCCACGC<br>R：CTTTGTCATTGTCCCACTTG	*Oreochromis niloticus*
MHCII	COP9 signalosome complex subunit 7a-like	F：ACCTCTTTGCCTATGGAAC<br>R：TGCTGGAGGAGCAGTGA	*Oreochromis niloticus*
MPO-d	eosinophil peroxidase-like	F: ACCACCAATGACGCTAAT<br>R: GTAGAGTCTCTCTCCATCCC	*Oreochromis niloticus*
MR	macrophage mannose receptor 1-like	F: TCTCCCAGCTCCTGATTG<br>R: CTCCATGCTGAGGATGGAAA	*Oreochromis niloticus*
MR-d	L-rhamnose-binding lectin CSL2-like	F: CGTAAACCTTGTTCGTAACTCTAA<br>R: TACACAAAGATAACCTGTCCTACATCA	*Oreochromis niloticus*

NFY	nuclear transcription factor Y subunit alpha-like	F: TGCCAACACCAACACTACAAA	*Oreochromis niloticus*
		R: TCCTCCTCCAGCATCTCA	
Piscidin	Piscidin-like antimicrobial peptide precursor	F: CTCTTTCTTGTGTTGTCGC	*Epinephelus coioides*
		R: GTCCAGCTCTTCCATGC	
PLG	plasminogen	F：CATGGACCTGTGCAAACTA	*Perca flavescens*
		R：CATGTTTTGCCAGATTCAGTTAC	
SERPING1	minus strand　C1 inhibitor	F：CCCTCATAATATGAATCTGAGCG	*Larimichthys crocea*
		R：AAATGTTGGATCTTATTGTTGGTC	
TAPBP	tapasin-like	F: AACAGCCGCTTAGGACA	*Oreochromis niloticus*
		R: TTTATATCCAGGCTGACGGAGA	
TfR	Transferrin receptor 1a	F: GATGTTATCACCGTGGAAGTC	*Danio rerio*
		R: CAACTGTCGATGTGGCAA	
TUBB	Tubulin, beta 5	F: CTGCTCATCAGCAAGATCC	*Danio rerio*
		R: CCTCATTGTCAATACAGTAGGTTT	

Supplemental Table 2: Gene ontology analysis of *Epinephelus coioides* larvae

Gene Ontology term	Cluster frequency	Genome frequency of use	Corrected P-value
Cellular component			
myosin complex	45 out of 1129 genes, 4.0%	162 out of 28794 genes, 0.6%	1.46E-23
actin cytoskeleton	79 out of 1129 genes, 7.0%	656 out of 28794 genes, 2.3%	1.62E-16
contractile fiber	59 out of 1129 genes, 5.2%	501 out of 28794 genes, 1.7%	1.28E-11
extracellular region	106 out of 1129 genes, 9.4%	1483 out of 28794 genes, 5.2%	3.55E-07
myofibril	43 out of 1129 genes, 3.8%	433 out of 28794 genes, 1.5%	6.02E-06
sarcomere	36 out of 1129 genes, 3.2%	327 out of 28794 genes, 1.1%	6.05E-06
contractile fiber part	36 out of 1129 genes, 3.2%	332 out of 28794 genes, 1.2%	8.94E-06
cytoskeleton	173 out of 1129 genes, 15.3%	3012 out of 28794 genes, 10.5%	3.39E-05
extracellular region part	92 out of 1129 genes, 8.1%	1346 out of 28794 genes, 4.7%	3.53E-05
striated muscle thin filament	9 out of 1129 genes, 0.8%	25 out of 28794 genes, 0.1%	5.28E-05
cytoskeletal part	108 out of 1129 genes, 9.6%	1724 out of 28794 genes, 6.0%	0.00021
non-membrane-bounded organelle	237 out of 1129 genes, 21.0%	4673 out of 28794 genes, 16.2%	0.00230
Molecular function			
hydrolase activity	283 out of 1138 genes, 24.9%	5158 out of 28380 genes, 18.2%	2.08E-06
cytoskeletal protein binding	101 out of 1138 genes, 8.9%	1398 out of 28380 genes, 4.9%	2.80E-06
hydrolase activity, acting on acid anhydrides	132 out of 1138 genes, 11.6%	2114 out of 28380 genes, 7.4%	8.62E-05

pyrophosphatase activity	131 out of 1138 genes, 11.5%	2097 out of 28380 genes, 7.4%	9.39E-05
hydrolase activity, acting on acid anhydrides, in phosphorus-containing anhydrides	131 out of 1138 genes, 11.5%	2103 out of 28380 genes, 7.4%	0.00010
structural molecule activity	52 out of 1138 genes, 4.6%	613 out of 28380 genes, 2.2%	0.00013
nucleoside-triphosphatase activity	124 out of 1138 genes, 10.9%	1977 out of 28380 genes, 7.0%	0.00016
endopeptidase activity	44 out of 1138 genes, 3.9%	501 out of 28380 genes, 1.8%	0.00042
active transmembrane transporter activity	72 out of 1138 genes, 6.3%	1007 out of 28380 genes, 3.5%	0.00063
phosphorylase activity	5 out of 1138 genes, 0.4%	11 out of 28380 genes, 0.0%	0.01458
peptidase activity	66 out of 1138 genes, 5.8%	989 out of 28380 genes, 3.5%	0.01501
peptidase activity, acting on L-amino acid peptides	62 out of 1138 genes, 5.4%	912 out of 28380 genes, 3.2%	0.01503
transporter activity	155 out of 1138 genes, 13.6%	2861 out of 28380 genes, 10.1%	0.02337
oligopeptide transporter activity	4 out of 1138 genes, 0.4%	7 out of 28380 genes, 0.0%	0.03071
potassium-transporting ATPase activity	8 out of 1138 genes, 0.7%	38 out of 28380 genes, 0.1%	0.04100
potassium ion transmembrane transporter activity	8 out of 1138 genes, 0.7%	38 out of 28380 genes, 0.1%	0.04100
Biological process			
lucose metabolic process	42 out of 1105 genes, 3.8%	279 out of 27537 genes, 1.0%	2.39E-10
hexose metabolic process	48 out of 1105 genes, 4.3%	443 out of 27537 genes, 1.6%	7.72E-07
muscle system process	52 out of 1105 genes, 4.7%	520 out of 27537 genes, 1.9%	2.54E-06

small molecule metabolic process	215 out of 1105 genes, 19.5%	3657 out of 27537 genes, 13.3%	4.39E-06
protein activation cascade	11 out of 1105 genes, 1.0%	31 out of 27537 genes, 0.1%	2.49E-05
monosaccharide metabolic process	48 out of 1105 genes, 4.3%	493 out of 27537 genes, 1.8%	2.50E-05
muscle contraction	37 out of 1105 genes, 3.3%	328 out of 27537 genes, 1.2%	2.55E-05
complement activation	9 out of 1105 genes, 0.8%	20 out of 27537 genes, 0.1%	4.39E-05
alcohol metabolic process	69 out of 1105 genes, 6.2%	854 out of 27537 genes, 3.1%	4.72E-05
regulation of muscle contraction	17 out of 1105 genes, 1.5%	89 out of 27537 genes, 0.3%	0.00011
glucose catabolic process	21 out of 1105 genes, 1.9%	138 out of 27537 genes, 0.5%	0.00022
small molecule biosynthetic process	56 out of 1105 genes, 5.1%	665 out of 27537 genes, 2.4%	0.00025
regulation of muscle system process	19 out of 1105 genes, 1.7%	116 out of 27537 genes, 0.4%	0.00027
skeletal muscle contraction	13 out of 1105 genes, 1.2%	55 out of 27537 genes, 0.2%	0.00029
hexose catabolic process	21 out of 1105 genes, 1.9%	141 out of 27537 genes, 0.5%	0.00033
alcohol catabolic process	21 out of 1105 genes, 1.9%	141 out of 27537 genes, 0.5%	0.00033
monosaccharide catabolic process	21 out of 1105 genes, 1.9%	141 out of 27537 genes, 0.5%	0.00033
nucleobase, nucleoside and nucleotide biosynthetic process	45 out of 1105 genes, 4.1%	491 out of 27537 genes, 1.8%	0.0004
nucleobase, nucleoside, nucleotide and nucleic acid biosynthetic process	45 out of 1105 genes, 4.1%	491 out of 27537 genes, 1.8%	0.0004
humoral immune response	10 out of 1105 genes, 0.9%	32 out of 27537 genes, 0.1%	0.00044
purine nucleoside triphosphate biosynthetic process	27 out of 1105 genes, 2.4%	223 out of 27537 genes, 0.8%	0.00052

purine ribonucleotide biosynthetic process	29 out of 1105 genes, 2.6%	251 out of 27537 genes, 0.9%	0.00053
striated muscle contraction	18 out of 1105 genes, 1.6%	111 out of 27537 genes, 0.4%	0.00066
cellular carbohydrate catabolic process	21 out of 1105 genes, 1.9%	151 out of 27537 genes, 0.5%	0.00106
nucleotide biosynthetic process	42 out of 1105 genes, 3.8%	465 out of 27537 genes, 1.7%	0.00146
purine ribonucleoside triphosphate biosynthetic process	26 out of 1105 genes, 2.4%	221 out of 27537 genes, 0.8%	0.00149
carbohydrate catabolic process	22 out of 1105 genes, 2.0%	168 out of 27537 genes, 0.6%	0.00166
nucleoside triphosphate biosynthetic process	27 out of 1105 genes, 2.4%	237 out of 27537 genes, 0.9%	0.00175
voluntary skeletal muscle contraction	11 out of 1105 genes, 1.0%	45 out of 27537 genes, 0.2%	0.00178
twitch skeletal muscle contraction	11 out of 1105 genes, 1.0%	45 out of 27537 genes, 0.2%	0.00178
ribonucleotide biosynthetic process	29 out of 1105 genes, 2.6%	271 out of 27537 genes, 1.0%	0.00259
purine nucleotide biosynthetic process	37 out of 1105 genes, 3.3%	398 out of 27537 genes, 1.4%	0.00325
musculoskeletal movement	14 out of 1105 genes, 1.3%	78 out of 27537 genes, 0.3%	0.00359
multicellular organismal movement	15 out of 1105 genes, 1.4%	89 out of 27537 genes, 0.3%	0.00361
cellular nitrogen compound biosynthetic process	52 out of 1105 genes, 4.7%	655 out of 27537 genes, 2.4%	0.00396
ribonucleoside triphosphate biosynthetic process	26 out of 1105 genes, 2.4%	233 out of 27537 genes, 0.8%	0.00404
fast-twitch skeletal muscle fiber	7 out of 1105 genes, 0.6%	18 out of 27537 genes, 0.1%	0.0053

contraction			
system process	146 out of 1105 genes, 13.2%	2523 out of 27537 genes, 9.2%	0.00577
cellular carbohydrate metabolic process	58 out of 1105 genes, 5.2%	772 out of 27537 genes, 2.8%	0.00596
cardiac muscle tissue morphogenesis	14 out of 1105 genes, 1.3%	84 out of 27537 genes, 0.3%	0.00891
regulation of striated muscle contraction	9 out of 1105 genes, 0.8%	35 out of 27537 genes, 0.1%	0.01073
immune effector process	22 out of 1105 genes, 2.0%	204 out of 27537 genes, 0.7%	0.03947

Supplemental Table 3: KEGG pathway enrichment analysis of *Epinephelus coioides* larvae

List	Pathway	NGS with pathway annotation (1504)	All genes with pathway annotation (32664)	P value	Q value	Pathway ID
	Metabolism					
	**Carbohydrate metabolism**					
12	Glycolysis / Gluconeogenesis	25 (1.66%)	173 (0.53%)	4.10E-07	7.42E-06	ko00010
18	Starch and sucrose metabolism	21 (1.4%)	167 (0.51%)	2.98E-05	3.59E-04	ko00500
27	Fructose and mannose metabolism	16 (1.06%)	152 (0.47%)	0.001784423	1.43E-02	ko00051
35	Pentose phosphate pathway	10 (0.66%)	85 (0.26%)	0.005645537	3.45E-02	ko00030
37	Galactose metabolism	14 (0.93%)	146 (0.45%)	0.007629812	4.47E-02	ko00052
43	Amino sugar and nucleotide sugar metabolism	14 (0.93%)	171 (0.52%)	0.02701486	1.36E-01	ko00520
48	Pyruvate metabolism	9 (0.6%)	101 (0.31%)	0.04333364	1.93E-01	ko00620
	**Amino acid metabolism**					
26	Cysteine and methionine metabolism	14 (0.93%)	117 (0.36%)	0.000991208	8.27E-03	ko00270
33	Glycine, serine and threonine metabolism	10 (0.66%)	84 (0.26%)	0.005190363	3.41E-02	ko00260
	**Metabolism of other amino acids**					
38	Glutathione metabolism	11 (0.73%)	114 (0.35%)	0.01613945	9.22E-02	ko00480
	**Metabolism of cofactors and vitamins**					
47	Retinol metabolism	12 (0.8%)	149 (0.46%)	0.04291683	1.93E-01	ko00830

	Genetic Information Processing					
	**Translation**					
23	Aminoacyl-tRNA biosynthesis	16 (1.06%)	136 (0.42%)	0.000540022	5.09E-03	ko00970
	Cellular Processes					
	**Cell communication**					
9	Tight junction	112 (7.45%)	1224 (3.75%)	3.60E-12	8.68E-11	ko04530
	**Transport and catabolism**					
15	Phagosome	55 (3.66%)	651 (1.99%)	1.27E-05	1.84E-04	ko04145
	**Cell motility**					
16	Regulation of actin cytoskeleton	98 (6.52%)	1387 (4.25%)	1.81E-05	2.45E-04	ko04810
	Organismal Systems					
	**Circulatory system**					
1	Cardiac muscle contraction	108 (7.18%)	742 (2.27%)	1.81E-26	3.94E-24	ko04260
11	Vascular smooth muscle contraction	88 (5.85%)	988 (3.02%)	2.97E-09	5.86E-08	ko04270
	**Digestive system**					
2	Protein digestion and absorption	91 (6.05%)	575 (1.76%)	4.50E-25	4.88E-23	ko04974
10	Pancreatic secretion	70 (4.65%)	618 (1.89%)	4.61E-12	1.00E-10	ko04972
21	Vitamin digestion and absorption	17 (1.13%)	146 (0.45%)	0.000415018	4.29E-03	ko04977
24	Mineral absorption	24 (1.6%)	253 (0.77%)	0.000694997	6.28E-03	ko04978
28	Salivary secretion	39 (2.59%)	517 (1.58%)	0.001889861	1.46E-02	ko04970
29	Carbohydrate digestion and absorption	26 (1.73%)	316 (0.97%)	0.003268099	2.45E-02	ko04973

32	Fat digestion and absorption	15 (1%)	153 (0.47%)	0.004800051	3.26E-02	ko04975
45	Bile secretion	27 (1.8%)	403 (1.23%)	0.03405557	1.64E-01	ko04976
	**Immune system**					
5	Complement and coagulation cascades	60 (3.99%)	341 (1.04%)	3.13E-19	1.36E-17	ko04610
17	Antigen processing and presentation	20 (1.33%)	152 (0.47%)	2.36E-05	3.01E-04	ko04612
30	Hematopoietic cell lineage	21 (1.4%)	238 (0.73%)	0.003475879	2.51E-02	ko04640
	**Excretory system**					
19	Proximal tubule bicarbonate reclamation	17 (1.13%)	132 (0.4%)	0.00012283	1.40E-03	ko04964
46	Aldosterone-regulated sodium reabsorption	17 (1.13%)	228 (0.7%)	0.03537639	1.67E-01	ko04960
	**Endocrine system**					
31	Renin-angiotensin system	7 (0.47%)	45 (0.14%)	0.004232833	2.96E-02	ko04614
41	PPAR signaling pathway	22 (1.46%)	301 (0.92%)	0.02281113	1.21E-01	ko03320
	**Sensory system**					
49	Olfactory transduction	17 (1.13%)	234 (0.72%)	0.04347116	1.93E-01	ko04740
	**Human Diseases**					
	**Cardiovascular diseases**					
3	Viral myocarditis	86 (5.72%)	604 (1.85%)	1.19E-20	7.37E-19	ko05416
4	Hypertrophic cardiomyopathy (HCM)	124 (8.24%)	1091 (3.34%)	1.36E-20	7.37E-19	ko05410
7	Dilated cardiomyopathy	123 (8.18%)	1172 (3.59%)	1.19E-17	3.70E-16	ko05414
36	Arrhythmogenic right ventricular cardiomyopathy (ARVC)	39 (2.59%)	552 (1.69%)	0.005718025	3.45E-02	ko05412

	**Infectious diseases: Bacterial**					
6	Staphylococcus aureus infection	44 (2.93%)	206 (0.63%)	1.00E-17	3.62E-16	ko05150
40	Pathogenic Escherichia coli infection	31 (2.06%)	457 (1.4%)	0.02117175	1.15E-01	ko05130
	**Immune diseases**					
8	Systemic lupus erythematosus	42 (2.79%)	248 (0.76%)	2.68E-13	7.28E-12	ko05322
14	Autoimmune thyroid disease	14 (0.93%)	72 (0.22%)	4.41E-06	6.84E-05	ko05320
20	Graft-versus-host disease	10 (0.66%)	57 (0.17%)	0.000248344	2.69E-03	ko05332
25	Allograft rejection	10 (0.66%)	66 (0.2%)	0.000834373	7.24E-03	ko05330
34	Asthma	6 (0.4%)	36 (0.11%)	0.005607425	3.45E-02	ko05310
	**Neurodegenerative diseases**					
13	Prion diseases	28 (1.86%)	225 (0.69%)	1.88E-06	3.14E-05	ko05020
	**Endocrine and metabolic diseases**					
22	Type I diabetes mellitus	12 (0.8%)	85 (0.26%)	0.000508753	5.02E-03	ko04940
44	Type II diabetes mellitus	21 (1.4%)	293 (0.9%)	0.03085919	1.52E-01	ko04930
	**Infectious diseases: Parasitic**					
39	Amoebiasis	39 (2.59%)	593 (1.82%)	0.01701984	9.47E-02	ko05146
42	Leishmaniasis	20 (1.33%)	269 (0.82%)	0.02469462	1.28E-01	ko05140

Supplemental Table 4: Summary of the variations in complement-related gene expression between controls and grouper challenged with *Vibrio alginolyticus*

Unigene	PREDICTED	1h	2h	3h	4h	5h	6h	7h	8h	12h	16h	24h	48h
A2M	Alpha-2-macroglobulin-like												
BF	Complement factor B-like			4.58	5.43	10.90	5.24	4.22	4.73	5.07			
C2r	C2r subcomponent-like		-2.22	-2.46									
C1q	Complement C1q-like protein 2								2.74				
C1r	Complement C1r subcomponent-like				2.39								
C1s	Haptoglobin-like				84.40	37.27	16.98	5.00	10.07				
C3	Complement component C3				3.21	4.40	3.74	2.23	3.21				
C4	Complement C4-like				2.13	4.01		2.45	3.60			4.11	

C4BP	Beta-2-glycoprotein 1 precursor					2.69			1.94	8.20		6.63	
C5	Complement C5								3.79	3.92	2.93	8.55	
C6	Complement component C6-like				10.90	16.08	19.77	22.38	14.82	23.19	9.81	16.39	
C6-d	Spondin-2-like	1.50				-2.95	-2.65	-6.95					
C7	Complement component C7-like				16.45	30.06	20.44	28.00	22.57	19.81	12.92		
C8	Complement component C8 beta					3.45			2.23	9.40	5.19	9.37	
C8-d	Complement component C8 alpha chain-like				2.48	3.48	2.55	3.86	4.05	6.83	2.74	9.04	
C9	Complement component C9				6.50	5.65	2.96	4.20	3.81	5.26	2.61	5.12	
DAF	Decay accelerating factor												

MASP1/2	Mannan-binding lectin serine protease 1		-1.81	-2.12	-1.84								
HF	Complement regulatory plasma protein					7.41	4.84	3.63	8.24				
IF	Complement factor I-like		11.83	11.23	16.61	16.71	7.55	5.13	4.47	7.20	2.36		
PLG	Ca2+-dependent complex C1R/C1S subunit	3.39						-3.12					
SERPING1	Minus strand C1 inhibitor												

Supplemental Table 5: Summary of the variations in phagocytosis-related gene expression between controls and grouper challenged with *Vibrio alginolyticus*

Unigene	PREDICTED	1h	2h	3h	4h	5h	6h	7h	8h	12h	16h	24h	48h
αVβ3	Integrin, alpha V												
αVβ3-2	Integrin beta-3-like												
αVβ5	Protein HEG-like					2.57							
Calnexin	Calnexin-like							1.83					
Cathepsin	Cathepsin L precursor					3.95	2.93	89.3					
Collectins	Lactose-binding lectin l-2-like						20.48						
DCSIGN	Type II antifreeze protein I											4.85	1.65
DCSIGN2	Nattectin												
Dynein	Cytoplasmic dynein 1 heavy chain 1-like isoform 2												

EEA1	Early endosome antigen 1										2.07	3.48	
F-actin	CDH1-D									1.37			
F-actin-d	Beta-centractin-like												
HSP70	Heat shock 70 kDa protein 14-like						1.81						
Ii-d	Invariant chain-like protein			1.71									
LAMP	Lysosomal membrane glycoprotein 2 precursor							2.05	2.54				
MHCI	Beta-centractin-like			1146.74									
MHCII	COP9 signalosome complex subunit 7a-like												
MPO-d	Eosinophil peroxidase-like										-4.23		

MR	Macrophage mannose receptor 1-like					7.95	2.53	26.32	13.92				
MR-d	L-rhamnose-binding lectin CSL2-like												
NFY	Nuclear transcription factor Y subunit alpha-like												
TAPBP	Tapasin-like					2.23	1.70						
TfR	Transferrin receptor 1a				2.98								
TUBB	Tubulin, beta 5								-1.33		-1.51		

Supplemental Table 6: Summary of the variations in antimicrobial peptide gene expression between controls and grouper challenged with *Vibrio alginolyticus*

Unigene	PREDICTED	1h	2h	3h	4h	5h	6h	7h	8h	12h	16h	24h	48h
Hepcidin	Hepcidin-like antimicrobial peptide precursor				141.48	1924.44	467.00	6385.32	4798.65				
LAP2	Liver-expressed antimicrobial peptide 2-like												
Piscidin	Piscidin-like antimicrobial peptide precursor								-1.96				
