# Supplementary figures and images for: Transcriptome analysis of the effect of Vibrio alginolyticus infection on the innate immunity-related complement pathway in Epinephelus coioides
Source: BMC Genomics. 2014 Dec 13;15(1):1102. doi: 10.1186/1471-2164-15-1102 (PMC4407539; doi:10.1186/1471-2164-15-1102)

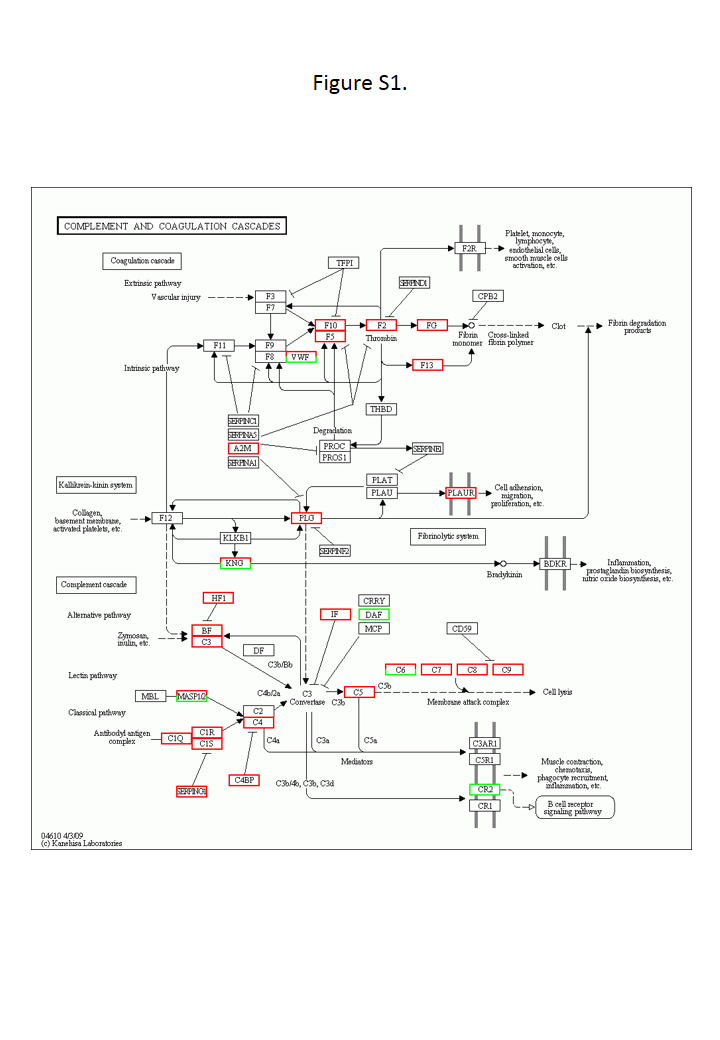

Supplement: Supplementary file 2 — Additional file 2: Figure S1.: Complement and coagulation cascades signal pathway. Enrichment analysis of DEGs from the KEGG database; red borders indicate up-regulated genes, green borders indicate down-regulated genes, and red/green borders indicate genes that are both up- and down-regulated at different times. (TIFF 233 KB) [file 12864_2014_6984_MOESM2_ESM.tiff]

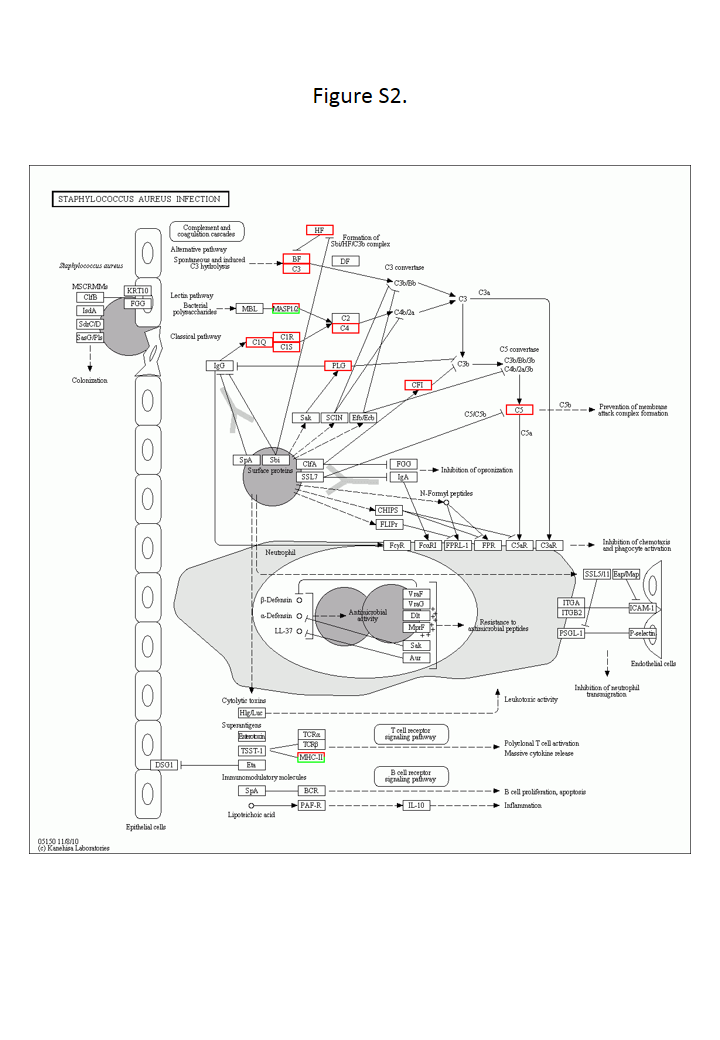

Supplement: Supplementary file 3 — Additional file 3: Figure S2.: Staphylococcus aureus infection signal pathway. Enrichment analysis of DEGs from the KEGG database; red borders indicate up-regulated genes, green borders indicate down-regulated genes, and red/green borders indicate genes that are both up- and down-regulated at different times. (TIFF 280 KB) [file 12864_2014_6984_MOESM3_ESM.tiff]

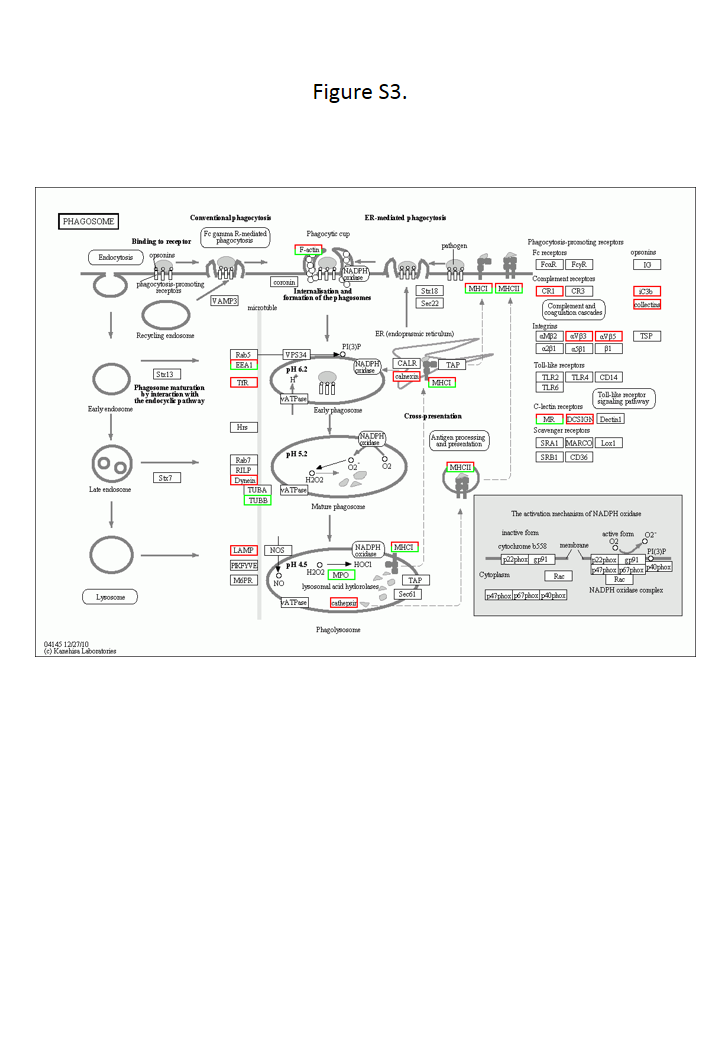

Supplement: Supplementary file 4 — Additional file 4: Figure S3.: Phagosome signal pathway. Enrichment analysis of DEGs from the KEGG database; red borders indicate up-regulated genes, green borders indicate down-regulated genes, and red/green borders indicate genes that are both up- and down-regulated at different times. (TIFF 259 KB) [file 12864_2014_6984_MOESM4_ESM.tiff]

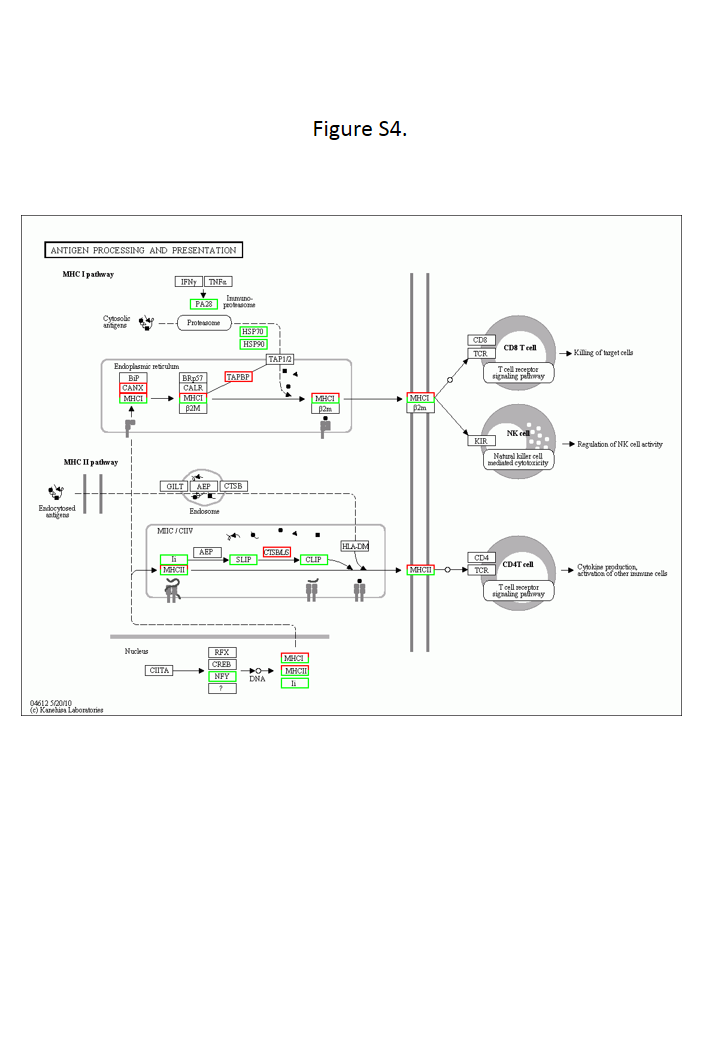

Supplement: Supplementary file 5 — Additional file 5: Figure S4.: Antigen processing and presentation signal pathway. Enrichment analysis of DEGs from the KEGG database; red borders indicate up-regulated genes, green borders indicate down-regulated genes, and red/green borders indicate genes that are both up- and down-regulated at different times. (TIFF 177 KB) [file 12864_2014_6984_MOESM5_ESM.tiff]

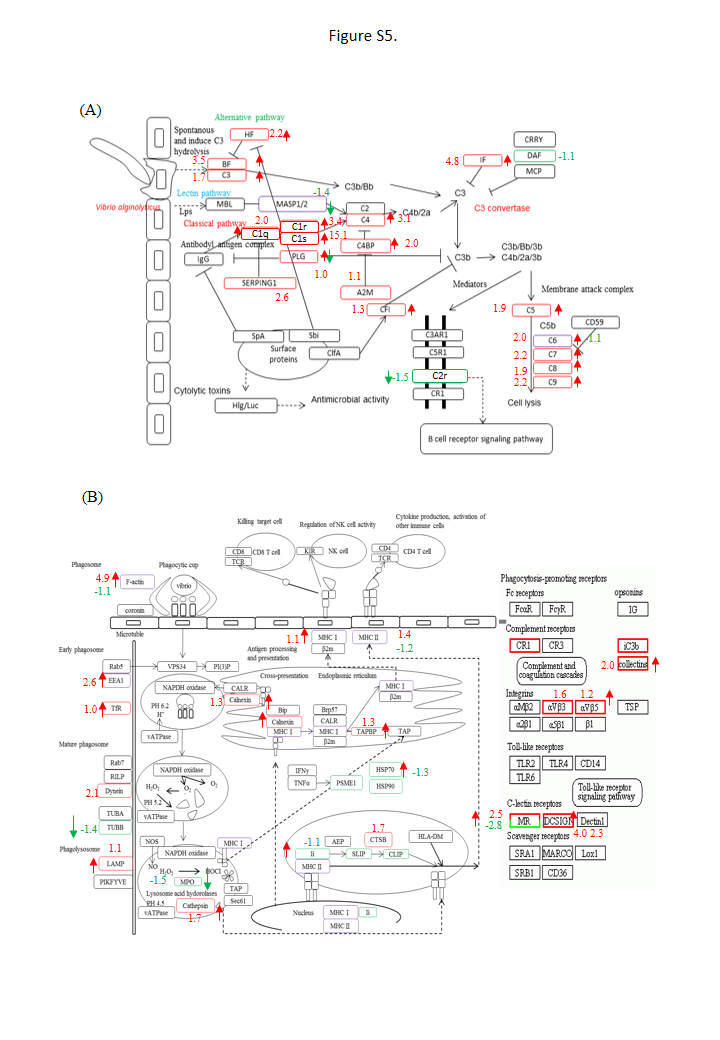

Supplement: Supplementary file 6 — Additional file 6: Figure S5.: Hypothetical model for the response of grouper larvae to Vibrio alginolyticus infection, as predicted by KEGG analysis. (A) Complement pathway. (B) Phagocytosis pathway. Red borders indicate up-regulated genes, green borders indicate down-regulated genes, and purple borders indicate genes that are both up- and down-regulated at different times. Red arrows indicate increased RNA expression and green arrows indicate decreased RNA expression. Numbers adjacent to borders are the log2 ratio of significantly affected genes. (TIFF 287 KB) [file 12864_2014_6984_MOESM6_ESM.tiff]
